# SARS-CoV-2: pathogenesis, therapeutics, variants, and vaccines

**DOI:** 10.3389/fmicb.2024.1334152

**Published:** 2024-06-13

**Authors:** Xi Li, Ze Mi, Zhenguo Liu, Pengfei Rong

**Affiliations:** ^1^Department of Radiology, The Third Xiangya Hospital, Central South University, Changsha, China; ^2^Department of Infectious Disease, The Third Xiangya Hospital, Central South University, Changsha, China

**Keywords:** SARS-CoV-2, COVID-19, immunity, cytokine storm, variants, treatment, vaccine

## Abstract

Coronavirus disease 2019 (COVID-19), caused by severe acute respiratory syndrome coronavirus 2 (SARS-CoV-2), emerged in December 2019 with staggering economic fallout and human suffering. The unique structure of SARS-CoV-2 and its underlying pathogenic mechanism were responsible for the global pandemic. In addition to the direct damage caused by the virus, SARS-CoV-2 triggers an abnormal immune response leading to a cytokine storm, culminating in acute respiratory distress syndrome and other fatal diseases that pose a significant challenge to clinicians. Therefore, potential treatments should focus not only on eliminating the virus but also on alleviating or controlling acute immune/inflammatory responses. Current management strategies for COVID-19 include preventative measures and supportive care, while the role of the host immune/inflammatory response in disease progression has largely been overlooked. Understanding the interaction between SARS-CoV-2 and its receptors, as well as the underlying pathogenesis, has proven to be helpful for disease prevention, early recognition of disease progression, vaccine development, and interventions aimed at reducing immunopathology have been shown to reduce adverse clinical outcomes and improve prognosis. Moreover, several key mutations in the SARS-CoV-2 genome sequence result in an enhanced binding affinity to the host cell receptor, or produce immune escape, leading to either increased virus transmissibility or virulence of variants that carry these mutations. This review characterizes the structural features of SARS-CoV-2, its variants, and their interaction with the immune system, emphasizing the role of dysfunctional immune responses and cytokine storm in disease progression. Additionally, potential therapeutic options are reviewed, providing critical insights into disease management, exploring effective approaches to deal with the public health crises caused by SARS-CoV-2.

## 1 Introduction

Severe acute respiratory syndrome coronavirus 2 (SARS-CoV-2) is a novel coronavirus (Tay et al., 2020; van Eijk et al., 2021) that has caused a pandemic of acute respiratory syndrome in humans following the outbreaks of Severe Acute Respiratory Syndrome Coronavirus (SARS-CoV) and Middle East Respiratory Syndrome Coronavirus (MERS-CoV; Zhou et al., 2020). Since the outbreak of COVID-19, over 600 million people have been confirmed to have tested positive for the virus, and more than 6 million patients have died.^1^ SARS-CoV-2 shares all typical structural features of other coronaviruses (Liang et al., 2020). However, compared with SARS-CoV, SARS-CoV-2 has certain structural changes that render it more infectious.

Similar to SARS-CoV, SARS-CoV-2 enters the cells by binding to its primary functional receptor, angiotensin-converting enzyme 2 (ACE2; Hashimoto et al., 2012; Varga et al., 2020), which is widely distributed in epithelial cells and in endothelial cells of tissues and organs throughout the body (Bourgonje et al., 2020; Yan et al., 2020). Both the tissue distribution of ACE2 and the effect of SARS-CoV-2 infection on the physiological functions of ACE2 determine, to a certain extent, the clinical features of the disease (Bourgonje et al., 2020).

COVID-19 cases present a wide range of clinical manifestations, ranging from mild symptoms such as dry cough, fever, and sore throat to severe acute respiratory distress syndrome (ARDS), multi-organ failure (MOF), and even death. The severity of COVID-19 is associated with the host immune response to SARS-CoV-2 (Huang et al., 2020; Tay et al., 2020). An effective immune response is crucial for viral clearance, while a dysfunctional response leads to viral persistence and excessive production of inflammatory factors, resulting in cytokine storm and disease progression. Therefore, understanding the interaction between SARS-CoV-2 and the host immune system, as well as identifying and characterizing the abnormal immune response induced by the virus, are essential for developing effective therapies.

Different disease stages in COVID-19 should be managed with tailored treatment options based on the pathogenic characteristics of SARS-CoV-2. During the early phase of the disease course, inhibiting viral entry and replication is critical, whereas in the late phase of the disease with high inflammatory status, measures to alleviate or control the inflammatory response should be applied (Schurink et al., 2020; Kim et al., 2021). Here we briefly overview the structural features of SARS-CoV-2 and its interaction with ACE2, elucidate some of the progress in understanding the associations of innate immunity, adaptive immunity and the cytokine storm with the immunopathogenesis of COVID-19, discuss the emerging variants of concern/interest (VOC/I) and several potential therapeutic options, outline the efficacy of different vaccines against SARS-CoV-2 and its variants. We provide insights into the treatment and management of the disease, exploring both individualized treatment strategies and the development of effective vaccines for future public health crises caused by coronaviruses.

## 2 Structure of SARS-CoV-2

The SARS-CoV-2 virus particles exhibit a spherical structure with a diameter of ~65–125 nm, and the spike (S) protein located on the viral surface gives the viruses a crown-like (“corona”) appearance, from which they acquired their name (Liang et al., 2020; Shereen et al., 2020). Enveloped coronaviruses possess the largest single-stranded, positive-sense RNA genome, with a length of approximately 26–32 kilobases (de Wit et al., 2016; Shereen et al., 2020; Walls et al., 2020). Two-thirds of the coronaviral genome encodes non-structural proteins, including RNA-dependent RNA polymerase (RdRp), proteases and helicase, which are responsible for viral replication; the 3′-end of the genome encodes the four main structural proteins of the coronavirus particles, namely the S, membrane (M), envelope (E) and nucleocapsid (N) proteins, as well as several accessory proteins that interfere with the host innate immune response (Pyrc et al., 2006; de Wit et al., 2016).

### 2.1 Spike protein

The S protein of SARS-CoV-2 has a trimeric structure and is located on the viral surface, giving the virus a “crown-like” appearance and serving as the major antigen that the host immune response targets (Liang et al., 2020; Shereen et al., 2020; Walls et al., 2020). SARS-CoV-2 infects host cells by binding to ACE2 through the S protein, which consists of two subunits, S1 and S2. The receptor-binding domain (RBD) of S1 binds receptors on the surface of host cells, allowing virus particles to attach to cells, while S2 promotes membrane fusion and the entry of RNA genes (Walls et al., 2020; Wrapp et al., 2020). Although the RBDs of SARS-CoV and SARS-CoV-2 share 72% similarity in their amino acid sequences (Tay et al., 2020), computational modeling and biophysical measurements have shown that SARS-CoV-2 RBD binds to ACE2 with higher affinity than the RBD of SARS-CoV (Wrapp et al., 2020). This may be due to the replacement of Val 404 in the SARS-CoV RBD with Lys 417 (a polar amino acid) in the SARS-CoV-2 RBD, resulting in tighter binding to ACE2 (Yan et al., 2020). However, the SARS-CoV-2 RBD is less exposed than the SARS-CoV RBD and is mostly found in a “lying-down” state that cannot bind to ACE2, which may be one of the immune evasion mechanisms of SARS-CoV-2 (Yuan et al., 2017; Shang et al., 2020). In addition, the S protein of SARS-CoV-2 contains a furin-like cleavage site that is absent in SARS-CoV, which enhances viral fusion with host cell membranes and probably contributes to the increased infectivity of SARS-CoV-2 compared to SARS-CoV (Walls et al., 2020; Wrapp et al., 2020).

### 2.2 M, E, and N proteins

The M protein of SARS-CoV-2 exists as a dimer and is the most abundant protein in the virus. It maintains the viral skeleton, transports nutrients across the membrane, promotes the release of nascent viral outgrowth, and plays a central role in viral assembly (Neuman et al., 2011). The E protein of SARS-CoV-2 is an ion channel that is essential for virus-host interactions and is associated with virulence. Moreover, the E protein participates in viral assembly and release (Nieto-Torres et al., 2014; Liang et al., 2020). Finally, the LKR region (also called SR) of the N protein of SARS-CoV-2 is a serine- and arginine-rich sequence that assists in cell signaling processes (You et al., 2005; Hurst et al., 2009; McBride et al., 2014).

### 2.3 Accessory proteins

The SARS-CoV-2 genome encodes different accessory proteins, including ORF3a, ORF3b, ORF6, ORF7a, ORF7b, ORF8, ORF9b, ORF9c, and ORF10, that can play key roles in the viral pathogenesis and immune evasion processes (Zandi et al., 2022). Therefore, these accessory proteins could be considered potential drug targets.

## 3 Cell entry of SARS-Cov-2

### 3.1 ACE2 receptor

**Figure 1:**
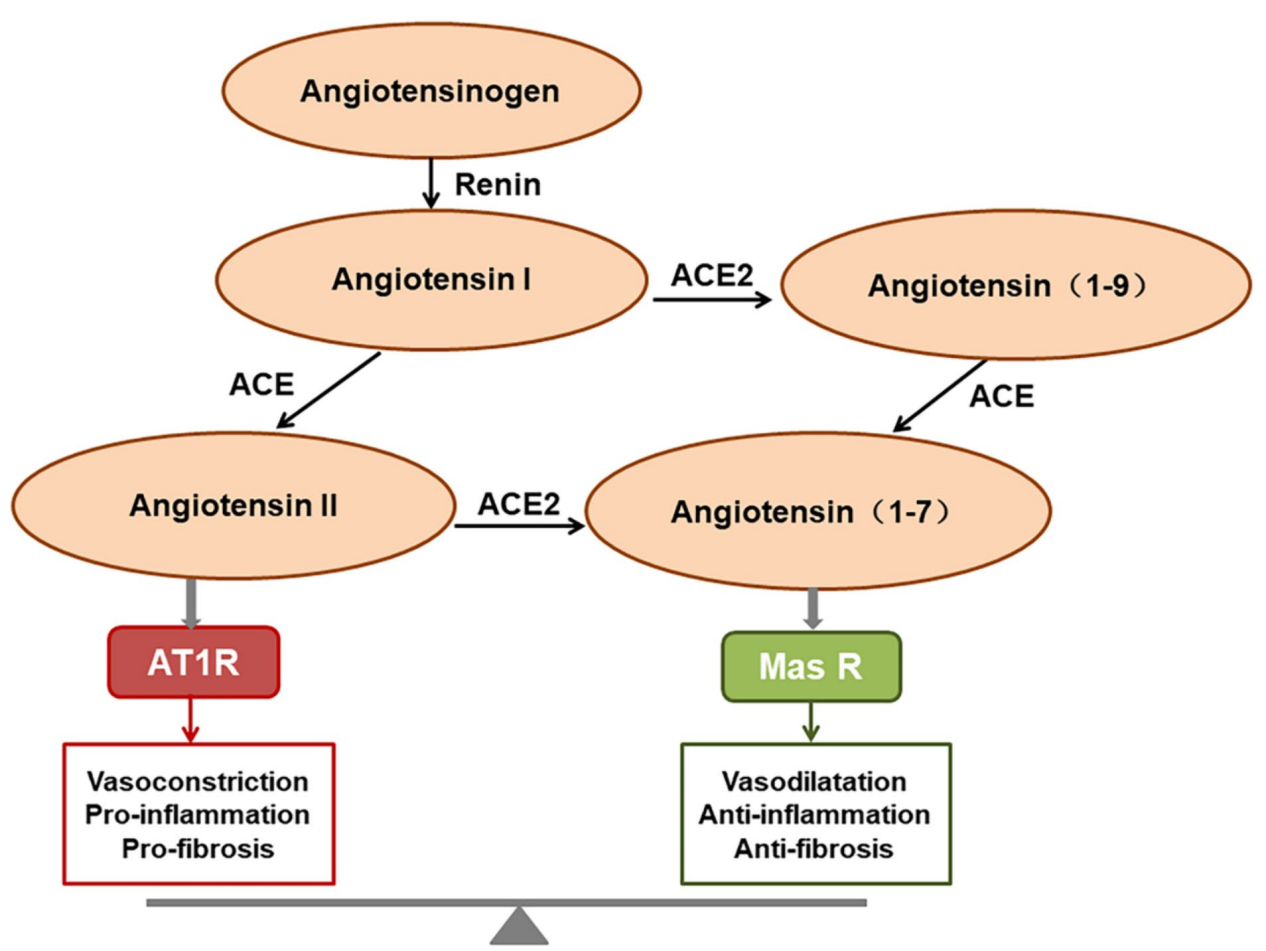
The function of the renin-angiotensin system (RAS). Angiotensin II predominantly exerts its role via activating the AT1 receptor, whereas angiotensin (1-7) [Ang (1-7)] binds to the Mas receptor to exert the opposing effects. The final homeostatic effect of the RAS depends on the balance of the angiotensin-converting enzyme (ACE)/angiotensin II (Ang II)/AT1R axis with the ACE2/Ang (1-7)/Mas receptor axis.

ACE2 is a homolog of angiotensin converting enzyme (ACE; Bourgonje et al., 2020), and plays a critical role in the renin-angiotensin system (RAS). ACE2 cleaves angiotensin II (Ang II) into angiotensin 1-7 (Ang1-7), which binds to the G protein-coupled receptor Mas, inhibiting the p38 MAPK and nuclear factor kappa B (NF-κB) signaling pathways, exerting expanded tubulars, anti-inflammatory, and anti-fibrotic effects (de Carvalho Santuchi et al., 2019; Magalhaes et al., 2020; South et al., 2020). The ACE2/Ang (1-7)/Mas receptor axis therefore functions in opposition to the ACE/Ang II/AT1 receptor axis (Bourgonje et al., 2020; Figure 1). ACE2 is the functional receptor for SARS-CoV-2 and SARS-CoV. One study demonstrated that SARS-CoV infection resulted in the downregulation of ACE2, which contributed to disease severity by disrupting the renin angiotensin aldosterone system in a rodent model (Kuba et al., 2005). The same is presumed for SARS-CoV-2, but the downregulation or exhaustion of ACE2 has not been demonstrated in the human SARS-CoV-2 model either *in vitro* or *in vivo*.

### 3.2 Cell entry mechanisms of SARS-CoV-2

SARS-CoV-2 enters host cells through two pathways, namely endocytic pathway and transmembrane serine protease 2 (TMPRSS2)-mediated surface pathway (Nazerian et al., 2021; van Eijk et al., 2021; Zhao et al., 2021). When SARS-CoV-2 binds to ACE2 through the RBD of the S protein, the structure of the S protein changes, allowing TMPRSS2 and cathepsins on the cell surface to cleave the S protein. Subsequently, proteolytic processing at the S1/S2 boundary and the S2 cleavage site removes the covalent connection of the two functional subunits, leading to the dissociation of S1 and facilitating the exposure of the S2 subunits’ domain, which initiates the fusion reaction by inserting of the hydrophobic fusion peptide into the cell membrane (Hoffmann et al., 2020; Walls et al., 2020; Wrapp et al., 2020). After binding, the virus becomes enveloped within endosome formed by the cell membrane and enters the cell through endocytosis. The early endosomes subsequently mature and acidify to form late endosomes, which ultimately develop into lysosomes. In lysosomes, lysosomal cathepsins facilitate the fusion of viral particles with the lysosomal membrane. Fusion between viral and cellular membranes forms a fusion pore through which viral RNA is released into the cytoplasm of the host cell for replication (Nazerian et al., 2021; Jackson et al., 2022). SARS-CoV-2 can also enter the cell via the TMPRSS2-dependent direct fusion of the viral envelope with the cell membrane, but the endocytic pathway is considered the main route (Heurich et al., 2014; van Eijk et al., 2021). Additionally, pre-activation of the protein convertase furin enhances the entry of SARS-CoV-2 into certain target cells, especially those with relatively low expression levels of TMPRSS2 and/or lysosomal cathepsins, contributing to increased infectivity (Shang et al., 2020).

## 4 Immune response against SARS-CoV-2 and immune defects

### 4.1 Innate immune response

#### 4.1.1 Innate immune dysregulation

The innate immune response provides the first defense against pathogenic microorganisms, and plays an important role in resisting viral invasion and initiating an adaptive immune response. Upon entry into cells, the pathogen-associated molecular patterns (PAMPs) of SARS-CoV-2 are recognized by endosomal pattern recognition receptors (PRRs), such as Toll-like receptors, which trigger intracellular signaling cascades, leading to the recruitment of neutrophils, activation of macrophages, and transcriptional activation of interferon regulatory factor (IRF) genes (Kawai and Akira, 2011). Furthermore, PAMPs recognition can also activate the NF-κB pathway, which leads to the production and signal transduction of pro-inflammatory cytokines, such as tumor necrosis factor-α (TNF-α) and interleukin-6 (IL-6), driving the inflammatory response (Saha et al., 2010; Hadjadj et al., 2020; Kim et al., 2021).

Interferon (IFN) is an essential component of the innate immune response and plays a crucial role in combating viral infections. Recognition of viral infections by innate immune sensors activates type I and type III IFN response (Park and Iwasaki, 2020). Type I and type III IFNs then induce hundreds of antiviral effectors, or Interferon-Stimulated Genes (ISGs), to establish a cell-intrinsic state of viral resistance (Schneider et al., 2014; Park and Iwasaki, 2020). Several SARS-CoV-2 proteins, such as open reading frame 6 (ORF6) and ORF3b, have the ability to suppress the IFN response (Konno et al., 2020; Park and Iwasaki, 2020; Minkoff and tenOever, 2023). Studies showed that serum IFN activity in severe or critical COVID-19 patients was significantly low, with striking downregulation of ISGs (Blanco-Melo et al., 2020; Hadjadj et al., 2020), indicating an impaired IFN response. When the IFN response is insufficient to control initial viral replication, delayed IFN could lead to inflammation and lung injury (Lee J. S. et al., 2020; Park and Iwasaki, 2020; Domizio et al., 2022; Minkoff and tenOever, 2023). As infected cells die, inflammatory substances associated with the virus are released into the extracellular space, enabling nearby cells to induce an IFN response (Minkoff and tenOever, 2023). The sustained increase in the levels of type I IFNs in the late phase of the infection promotes the accumulation of monocytes-macrophages and the production of pro-inflammatory cytokines, resulting in lethal pneumonia with vascular leakage and impaired virus-specific T cell responses (Lee J. S. et al., 2020). Additionally, this delayed IFN response can lead to systemic inflammation, which is associated with adverse clinical outcomes (Park and Iwasaki, 2020; Domizio et al., 2022; Minkoff and tenOever, 2023).

**Figure 2:**
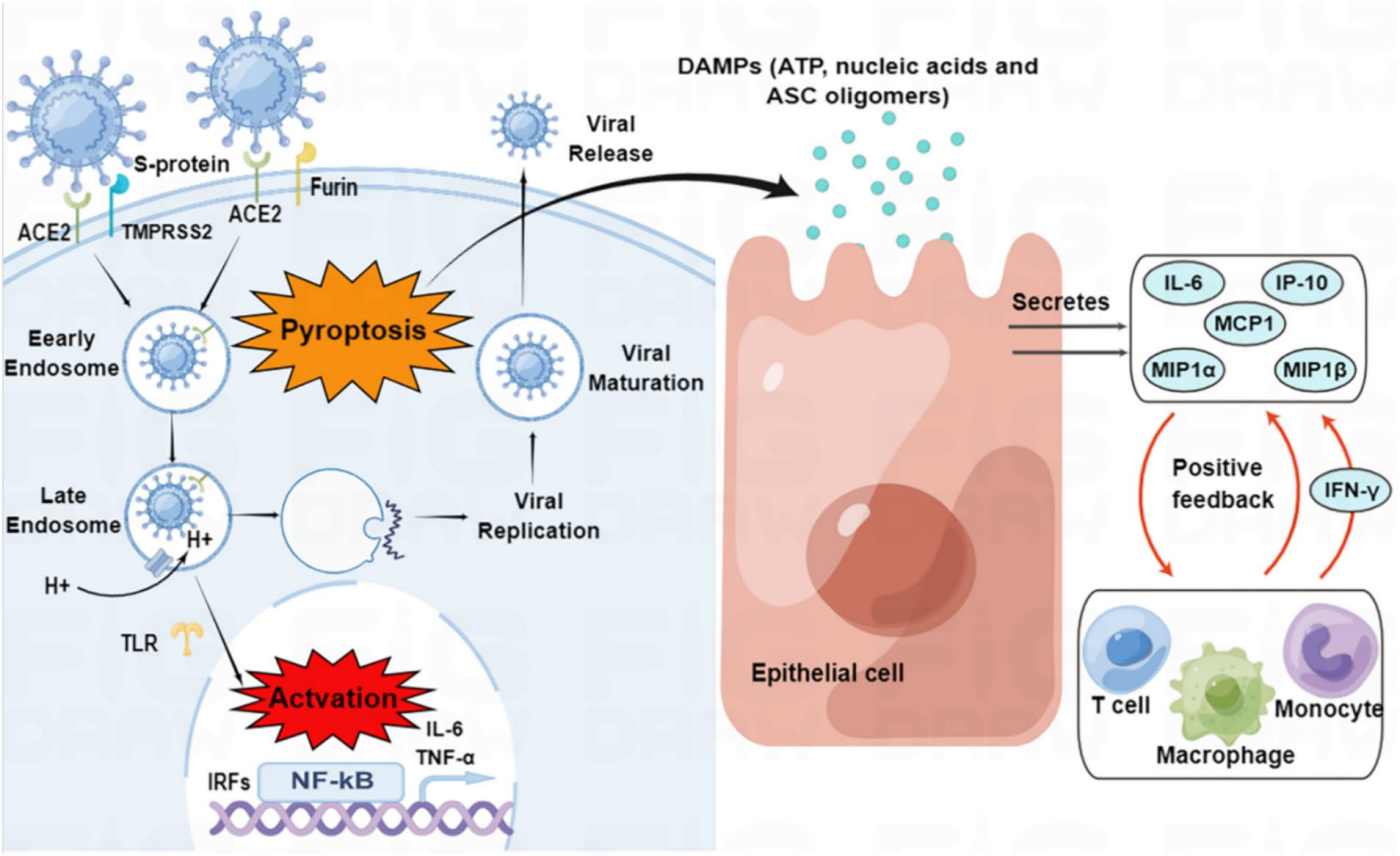
SARS-CoV-2 entry and immune activation. SARS-CoV-2 enters the cell by binding to angiotensin-converting enzyme 2 (ACE2) through transmembrane serine protease 2 (TMPRSS2)-enhanced endocytosis. Furin can also facilitate viral entry. The pathogen-associated molecular patterns (PAMPs) of SARS-CoV-2 are recognized by endosomal pattern recognition receptors, including Toll-like receptors (TLRs), which activate interferon regulatory factor (IRF) genes and nuclear factor kappa B (NF-κB). Pyroptosis of host cells releases damage-associated molecular patterns (DAMPs), which neighboring epithelial cells, endothelial cells, and alveolar macrophages recognize, triggering the production of pro-inflammatory cytokines and chemokines, such as interleukin (IL)-6, interferon γ-induced protein 10 (IP-10), macrophage inflammatory protein 1α (MIP1α), MIP1β and monocyte chemoattractant protein 1 (MCP1). These cytokines and chemokines attract monocytes, macrophages, and T cells to the site of infection, promoting further inflammation and establishing a pro-inflammatory feedback loop.

On the other hand, active virus replication and release cause host cells to undergo pyroptosis, releasing damage-associated molecular patterns (DAMPs) that neighboring epithelial cells, endothelial cells, and alveolar macrophages recognize, initiating the production of pro-inflammatory cytokines and chemokines, which attract monocytes, macrophages, and T cells to the site of infection, promoting further inflammation and establishing a pro-inflammatory feedback loop (Figure 2; Tay et al., 2020). Furthermore, increasing evidence suggests that overactivation of the complement system may be one of the pathogenic mechanisms of SARS-CoV-2 (Holter et al., 2020; Mastellos et al., 2020).

#### 4.1.2 The role of SARS-CoV-2 accessory proteins in innate immune response

SARS-CoV-2 accessory proteins have the capacity to regulate various aspects of host immunity, including stress response, autophagy, apoptosis, and innate immunity (Zandi et al., 2022). The majority of these accessory proteins act as interferon antagonists, including ORF6, ORF3b, ORF7a, ORF7b, ORF8, ORF9b, and ORF10, impeding the IFN response through diverse mechanisms. For instance, ORF6 prevents the phosphorylation of STAT to inhibit IFN activation, while polyubiquitination of ORF7a can suppress the IFN-I response by inhibiting STAT2 (Zandi et al., 2022). ORF10 has exhibited the ability to suppress the IFN-I signaling pathway by interacting with mitochondrial antiviral signaling protein (MAVS; Li X. et al., 2022; Zandi, 2022). Additionally, ORF3a and ORF3b modify crucial cellular processes such as apoptosis and autophagy (Wong and Perlman, 2022; Zandi et al., 2022). The ectodomain of ORF7 binds to CD14+ monocyte markers, potentially attenuating antigen-presenting cell (APC) function and leading to overexpression of proinflammatory cytokines. This interaction between ORF7a and monocytes suggests its potential role in recruiting monocytes to the lungs during COVID-19 (Zandi et al., 2022). The ORF8 protein downregulates the presentation of viral antigens via the class I major histocompatibility complex (MHC-I), thus dampening the host’s innate immune response (Zandi et al., 2022). Upregulation of ORF9b induces autophagy. ORF9b can also activate inflammasome as a means of evading immune responses (Zandi et al., 2022). Furthermore, the ORF9c accessory protein of SARS-CoV-2 interferes with antigen presentation, interferon signaling, and other immune and stress pathways in human lung epithelial cells (A549 cell line), suggesting its potential involvement in immune evasion processes. However, it should be noted that these experiments were performed using *in vitro* overexpression systems, and the expression of these proteins in virally infected cells within the context of an *in vivo* SARS-CoV-2 infection remains unclear (Zandi, 2022). Overexpression of ORF10 triggers the process of mitophagy by promoting the accumulation of LC3 in mitochondria (Li X. et al., 2022; Zandi, 2022). Both ORF3a and ORF7a have been reported to induce the expression of inflammatory cytokines through the activation of NF-κB signaling (Wong and Perlman, 2022).

### 4.2 Adaptive immune response

#### 4.2.1 Cellular immunity

SARS-CoV-2 parasitizes within host cells, relying on them for replication and survival, thus cellular immunity plays a crucial role in eliminating viral infection. However, an abnormal activation and differentiation of T cells occurs in severe cases of SARS-CoV-2 infection (Kalfaoglu et al., 2020). The first autopsy of COVID-19 revealed a substantial reduction in peripheral CD4+ or CD8+ T cell counts, while their status was hyper-activated (Xu Z. et al., 2020). The proportion of highly pro-inflammatory CCR6+ Th17 cells in the CD4+ T cell population was increased (Xu Z. et al., 2020). Patients with severe SARS-CoV-2 infection exhibited a significant decrease in the proportion of multifunctional CD4+ T cells compared to those with mild infection, suggesting that the function of CD4+ T cells had been compromised (Zheng et al., 2020). Furthermore, CD8+ T cells were found to have high concentrations of cytotoxic granules, with 31.6% of the cells being perforin-positive, 64.2% granulysin-positive, and 30.5% double positive for granulysin and perforin (Xu Z. et al., 2020). CD8+ T cells showed high granular enzyme cytotoxicity, with increased expression levels of immune checkpoint inhibitory receptors programmed cell death protein 1 (PD-1), cytotoxic T-lymphocyte-associated antigen 4 (CTLA-4) and T cell immunoreceptor with Ig and ITIM domains (TIGIT; Zheng et al., 2020). Following SARS-CoV-2 infection, the levels of CD38+ T cells expressing depletion markers were significantly increased (Wauters et al., 2021). These studies suggest that both elevated exhaustion levels and reduced functional diversity of T cells in severe cases of SARS-CoV-2 infection may be associated with the progression of COVID-19 patients (Zheng et al., 2020). Moreover, T cells in patients with severe COVID-19 express high levels of CD25, which produces the protease furin that facilitates the cell entry of SARS-CoV-2 (Kalfaoglu et al., 2020).

Lymphopenia is one of the main features of severe SARS-CoV-2 infection (Tay et al., 2020; van Eijk et al., 2021). The virus has been shown to induce programmed T cell death, such as apoptosis, contributing to lymphopenia. Reports of reduced peripheral blood T cell levels in patients suggest that T cells are stimulated to exit the blood and migrate to sites of infection to control the viral infection (Tay et al., 2020). van Eijk et al. (2021) proposed that abnormal innate immune responses, direct SARS-CoV-2 infection of T cells, virus-induced tissue damage of lymphatic organs, cytokine-induced apoptosis and pyroptosis of lymphocytes, macrophage activation syndrome (MAS)-associated hemophagocytosis, lymphocyte sequestration in lungs or other organs, and reduced bone marrow hematopoiesis can all contribute to COVID-19-associated lymphopenia. These explanations represent the most comprehensive understanding of lymphopenia associated with COVID-19 to date.

#### 4.2.2 Humoral immunity

Antigen-presenting cells (APCs) recognize antigens and stimulate the body’s humoral immunity via virus-specific B and plasma cells to produce neutralizing antibodies. Neutralizing antibodies prevent infection by blocking S protein interaction with ACE2 receptors and viral uncoating (Forchette et al., 2021). Antibody responses to SARS-CoV-2 typically arise within 4–8 days after symptom onset, and most patients seroconvert within the first 3 weeks (Liang et al., 2020; Tay et al., 2020; To KK et al., 2020). The production of IgM is an early and transient response to new antigens, while IgG predominates as a long-term antibody with a longer half-life and lower molecular weight, providing long-term protection and effective tissue penetration (Liang et al., 2020).

Antibodies play a crucial role as a fundamental component of protective immunity against pathogens. However, when an antibody against a pathogen—whether acquired by an earlier infection, vaccination or passive transfer—worsens its virulence through mechanisms dependent on antibodies, the severity of the disease in infected individuals or animals is enhanced. This phenomenon is known as antibody-dependent enhancement (ADE; Arvin et al., 2020). ADE has been documented to occur through two distinct mechanisms in viral infections. Generally, antibodies bind viruses to immune cells via Fcγ receptors on the cell surface and internalization of viruses typically results in their degradation. If antibody binding enhances the ability of viral proteins to enter target cells, or if the virus possesses the capability to evade destruction and produce more viruses after Fcγ receptor-mediated entry, the infection will be amplified. Additionally, if antibodies that bind viruses and Fcγ receptors on cells of the immune system lead to excessive release of cytokines, or if there is an excessive activation of complement due to the abundant formation of immune complexes between antibodies and viral proteins (antigens), it can result in increased disease severity (Arvin et al., 2020; Bournazos et al., 2020; Lee W. S. et al., 2020). ADE of disease is a general concern for the development of vaccines and antibody therapies, and it has been observed in various viruses such as respiratory syncytial virus and dengue virus (Lee W. S. et al., 2020). During the early stages of the COVID-19 pandemic, scientists indeed expressed concern about the potential occurrence of ADE of the disease. However, as research progressed and more data was accumulated, no definitive evidence has been found to support the existence of ADE in COVID-19.

## 5 Cytokine storm and organ involvement

### 5.1 Cytokine storm

**Figure 3:**
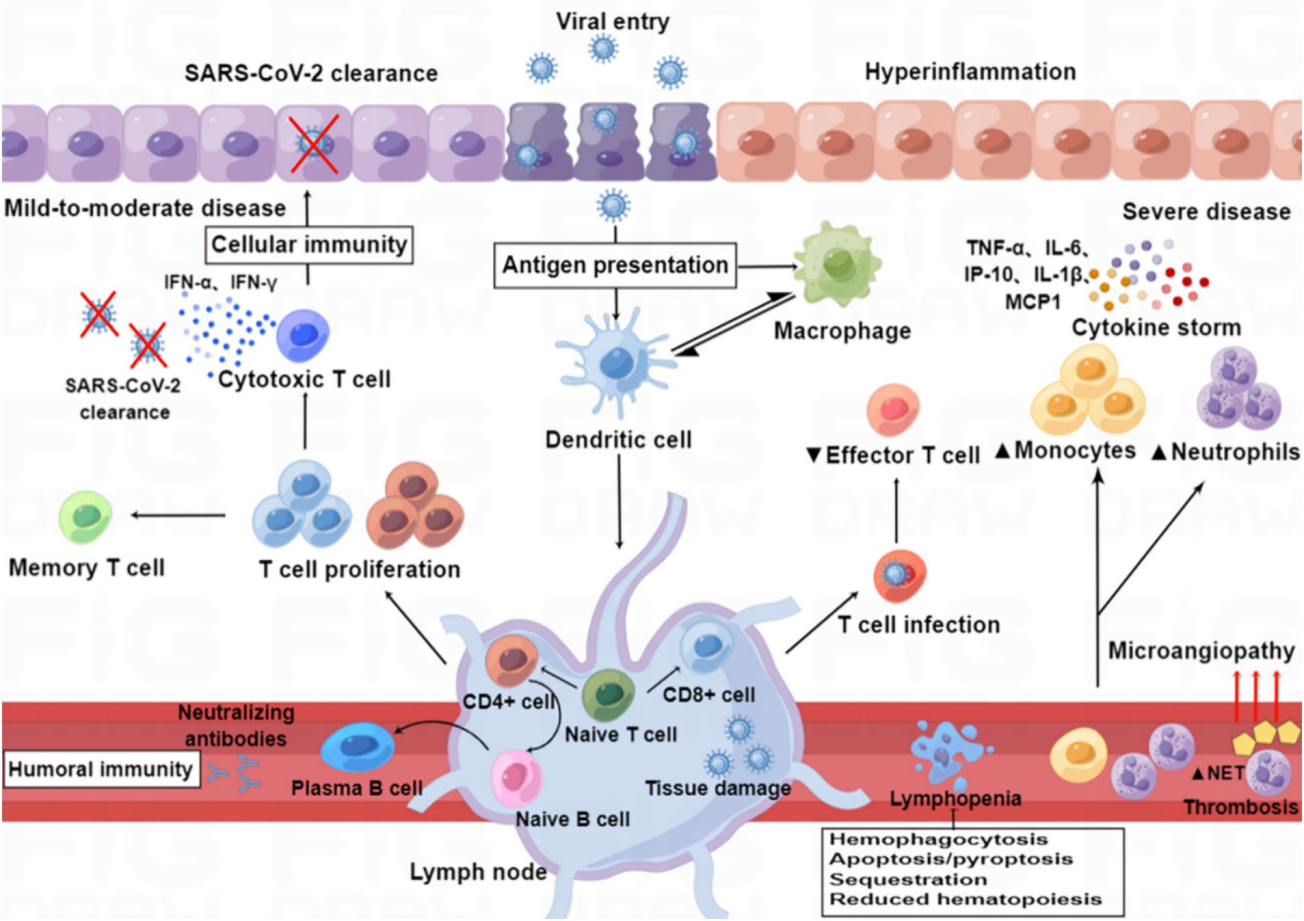
Immunopathology of SARS-CoV-2 infection. Patients with mild to moderate COVID-19 can clear the virus with a healthy immune response, while those with severe infection show immune dysfunction, characterized by relatively increased levels of neutrophils and monocytes and decreased levels of effector T lymphocytes. SARS-CoV-2 infection induces an excess of cytokines, which contribute to hyper-inflammation as constituents of the “cytokine storm” in severe disease (e.g., IL-6, IP-10, IL-1β, TNF-α, and MCP-1), whereas others are particularly important for viral clearance in mild to moderate disease (e.g., IFN-α and IFN-γ). SARS-CoV-2 infection also induces the release of neutrophil extracellular traps (NETs), and excessive formation of NETs leads to a strong pro-coagulant response. Lymphopenia may be caused by hemophagocytosis, reduced hematopoiesis and increased apoptosis/pyroptosis.

“Cytokine storm” is a condition of uncontrolled systemic hyper-inflammation caused by cytokine excess, which can lead to MOF, and even death (Yang et al., 2020; Kim et al., 2021). SARS-CoV-2 infection causes ACE2 receptor internalization, with a subsequent reduction in the plasma membrane levels of ACE2, resulting in overactivation of the ACE/Ang II/AT1 receptor axis and loss of ACE2/Ang1-7/Mas receptor axis, eventually leading to the overproduction of inflammatory factors (Mahmudpour et al., 2020; Verdecchia et al., 2020). A dysfunctional immune response also contributes to the cytokine storm (Kim et al., 2021). Macrophages are one of the main components of the immune system that are involved in creating the cytokine storm, and these are highly prone to polarization toward the M1 phenotype following SARS-CoV-2 infection, which leads to the release of excessive amounts of inflammatory factors (Nazerian et al., 2021). Aberrant activation of NF-κB caused by SARS-CoV-2 infection also leads to the expression of transcription factors that may drive this exacerbated inflammation (Hadjadj et al., 2020). Furthermore, the complement component C3a promotes differentiation of CD16-expressing highly cytotoxic T cells, which subsequently promotes the release of neutrophils and monocyte chemoattractants (Georg et al., 2022). The cytokine storm arises in part from an abnormal IFN response (Kim et al., 2021), which was supported by a study by Blanco-Melo et al. (2020), who performed transcriptome profiling of respiratory cell types to show that SARS-CoV-2 infection elicits exceptionally low IFN levels while inducing a robust pro-inflammatory cytokine response (Figure 3).

Previous studies have shown that various cytokines, including IL-1 family members, IL-6, IL-8, IL-10, TNF-α, IFN-γ, IFN-γ-induced protein 10 (IP-10), monocyte chemoattractant protein 1 (MCP1) and granulocyte colony-stimulating factor (GM-CSF), are elevated in severe SARS-CoV-2 infection (Huang et al., 2020; Mortaz et al., 2020; Kim et al., 2021). These pro-inflammatory cytokines play important roles in the acute phase of inflammation. IL-6 promotes the production of several acute-phase proteins, priming inflammatory responses (McGonagle et al., 2020a). GM-CSF links T-cell-driven acute pulmonary inflammation with an autocrine, self-amplifying cytokine loop, leading to monocyte and macrophage activation (Mortaz et al., 2020).

### 5.2 Organ involvement

#### 5.2.1 Lung involvement

The abnormal host response or immune system’s overreaction to SARS-CoV-2 can result in the production of extremely high levels of inflammatory cytokines, chemokines and free radicals, causing severe damage to multiple organs (Mortaz et al., 2020). The typical clinical manifestations of lung involvement include fever, dry cough and dyspnea (Bourgonje et al., 2020). Although most patients experience mild symptoms, a significant proportion of patients develop the more serious condition, namely viral pneumonia and ARDS (Huang et al., 2020). Autopsy studies show that the lung pathology is diffuse alveolar damage rather than diffuse alveolar hemorrhage, supporting the role of ARDS in COVID-19 mortality (Liu et al., 2020; Xu Z. et al., 2020). Cytokine storm may be a crucial mechanism underlying ARDS. Elevated levels of IL-1 in SARS-CoV-2 infection lead to the accumulation and activation of a large number of neutrophils in the lung, wherein oxygen free radicals, proteases and inflammatory mediators are released through a “respiratory burst,” causing damage to target cells, which eventually leads to alveolar cell loss, hyaline membrane formation and pulmonary edema, limiting lung gas exchange and causing respiratory distress and hypoxaemia (Tay et al., 2020; Ronit et al., 2021). The levels of C-X-C motif chemokine ligand 10 (CXCL10)/IP-10 and GM-CSF in patients with COVID-19 ARDS were also found to be elevated (Hue et al., 2020), which result in the perpetuation or further amplification of the inflammatory process, respectively. However, the role of cytokine storm in COVID-19-associated ARDS is still under debate since some studies have shown low pro-inflammatory cytokine levels compared to archetypical conditions associated with MAS (Leisman et al., 2020).

Smoking may increase the risk of ARDS due to the upregulation of ACE2 in the airways, which facilitates virus entry (Leung et al., 2020). Obesity may also be a risk factor for ARDS, since visceral fat is able to induce pro-inflammatory effects (Jose and Manuel, 2020). Poor chest wall elasticity and respiratory system compliance in obese patients lead to impaired lung function (Gao et al., 2021). Diabetic patients with elevated levels of IL-6 may have an increased risk of thrombosis that leads to blood-air barrier dysfunction (Gao et al., 2021), thereby increasing the risk of ARDS. Hypertension may also increase the risk of ARDS due to downregulation of ACE2/Ang(1-7) and upregulation of ACE/Ang II, leading to increased inflammatory responses (South et al., 2020). These comorbidities may contribute to the progression of COVID-19 patients to ARDS.

#### 5.2.2 Thromboembolic risk

SARS-CoV-2 infection can cause vascular changes such as severe endothelial injury, disrupted endothelial cell membranes, and widespread vascular thrombosis with microangiopathy, ultimately lead to multi-organ failure (Ackermann et al., 2020; Perico et al., 2021). Infection-mediated endothelial injury and endothelialitis, found in multiple vascular beds (including the lungs, kidney, heart, small intestine, and liver) in patients with COVID-19, can trigger excessive thrombin production, inhibit fibrinolysis, and activate complement pathways, initiating thromboinflammation and ultimately leading to microthrombi deposition and microvascular dysfunction (Gupta et al., 2020). However, the finding that viral RNA is rarely detectable in blood indicates that systemic endothelial dysfunction and vasculopathy in COVID-19 patients are not caused by a direct effect of the virus on endothelial cells. The hypercytokinaemia and the massive pro-inflammatory response of the host may contribute to endothelial dysfunction in COVID-19, particularly via the actions of IL-6 and TNF (Teuwen et al., 2020).

Excessive complement activation has been shown to cause intravascular coagulation (Noris et al., 2020). Activation of the complement system leads to endothelial cell injury and death with subsequent vascular denudation and exposure of the thrombogenic basement membrane, which drives activation of clotting cascades (Perico et al., 2021). In addition, the damage and inflammation in endothelial cells lead to the release of PAMPs and DAMPs, which are recognized by monocytes with expression of pattern PRRs, resulting in a high expression of tissue factor in them, thereby activating the extrinsic coagulation pathway (van Eijk et al., 2021). Furthermore, viral infection induces the release of neutrophil extracellular traps (NETs) composed of neutrophil-derived DNA and acetylated histones, which capture and kill invading pathogens as part of the innate immune. However, the excessive formation of NETs can cause a strong pro-coagulant response and are found in various organs of COVID-19 patients (Brinkmann et al., 2004; Middleton et al., 2020; Schurink et al., 2020; Borczuk, 2021). The presence of high levels of platelet factor 4 (PF4), IL-6 and IL-8 in the serum can induce the formation of NETs (Rossaint et al., 2014; Middleton et al., 2020). An increased formation of NETs was a potential biomarker of disease severity. Elevated D-dimer levels reflect vascular bed thrombosis with fibrinolysis (Tang et al., 2020a,b; McGonagle et al., 2020b). Antiphospholipid antibody levels are elevated in critical COVID-19 patients, increasing the risk of (micro)vascular thrombosis (MIYAKIS et al., 2006; Zhang Y. et al., 2020).

#### 5.2.3 Neurological involvement

Some COVID-19 patients may experience neurological symptoms such as headache, confusion, anosmia, taste disturbance, nausea, and vomiting (Mao et al., 2020). Anosmia is the most common and specific neurological presentation in COVID-19 patients. The infection of non-neuronal sustentacular cells in the olfactory mucosa by the SARS-CoV-2 and the subsequent local inflammation are associated with the anosmia (Minkoff and tenOever, 2023). Persistent anosmia in certain COVID-19 patients may be due to axon injuries and microvasculopathy in olfactory tissue, as suggested by higher mean axon pathology scores and microvasculopathy scores, lower axon density, and ultrastructural changes in olfactory axons (Ho et al., 2022). In addition, SARS-CoV-2 infection causes widespread downregulation of olfactory receptors (ORs) and of their signaling components (Zazhytska et al., 2022). However, whether olfactory neurons can be infected by SARS-CoV-2 remains controversial (de Melo et al., 2021; Khan et al., 2021).

Central nervous system (CNS) manifestations are the major forms of neurological involvement seen in COVID-19 patients (Mao et al., 2020). Systemic inflammation-induced neuroinflammation, consequent glial dysregulation and impairment of neural function represents a pathological mechanism of COVID-19 (Fernández-Castañeda et al., 2022). Autopsy findings showed hyperaemic and edematous brain tissue, neuronal degeneration, and activation of microglia with the formation of nodules (Mao et al., 2020; Schurink et al., 2020). Following mild respiratory COVID in mice, persistently impaired hippocampal neurogenesis, decreased oligodendrocytes, and myelin loss were evident together with elevated cerebrospinal fluid (CSF) cytokines/chemokines including CCL11. Concordantly, humans who experience persistent cognitive symptoms after COVID-19 exhibit elevated CCL11 levels (Fernández-Castañeda et al., 2022). In the brains of deceased COVID-19 patients, extensive inflammation was seen in the olfactory bulbs and medulla oblongata (Schurink et al., 2020). Furthermore, in the context of pro-inflammatory hypercoagulable state of COVID-19, acute cerebrovascular disease is also emerging as an important complication (Ellul et al., 2020). Viral RNA has been detected in the CSF and other brain tissues collected from patients who died from COVID-19 (Ellul et al., 2020; Matschke et al., 2020). However, the direct role of the virus in the neurological manifestations and the portal of entry of SARS-CoV-2 in the CNS remain highly debated (de Melo et al., 2021).

A novel neurological manifestation called COVID-19 hemiencephalitis has recently been observed. This condition is characterized by hyperintensity in one cerebral hemisphere on fluid-attenuated inversion recovery images and subsequent brain atrophy (Wang and Yin, 2024). Additionally, a study found an increased incidence of functional neurological disorders (FNDs) associated with long-COVID (Alonso-Canovas et al., 2023). Reports of neurological sequelae, commonly referred to as “brain fog,” are also becoming more prevalent. According to Greene et al., persistent localized blood–brain barrier (BBB) dysfunction and sustained systemic inflammation are key features of long COVID-related brain fog. These findings suggest that targeted regulation of BBB integrity may offer a new approach for managing patients with long-COVID (Greene et al., 2024).

## 6 Evolution and mutation

The Wuhan-Hu-1 is not the direct ancestor of all early new coronaviruses worldwide. It has resulted from three α mutations, consisting of two synonymous and one non-synonymous mutation in the ancestral strain (Kumar et al., 2021). However, the exact origin has not yet been determined. Studies indicate that SARS-CoV-2 may have entered the human population earlier than 2019 through unnoticed infections. During this period of cryptic transmission, the virus could have gradually acquired key mutations, including the RBD and furin cleavage site insertions, which facilitated its adaptation to humans (Zhang and Holmes, 2020). The acquisition of the furin cleavage site by SARS-CoV-2 and subsequent cleavage of the S protein is essential for human infection. The virus retained this cleavage site throughout the pandemic (Jackson et al., 2022).

SARS-CoV-2 has the ability to produce significant genomic changes (Davies et al., 2021; Harvey et al., 2021). In general, as viruses evolve to escape immune surveillance, they often undergo reduced fitness and become less infectious (Dazert et al., 2009; das et al., 2011; Sui et al., 2014; Shang et al., 2020). However, SARS-CoV-2 remains highly infectious despite being immune evasive, which may contribute to its widespread transmission (Chu et al., 2020; Sanche et al., 2020; Shang et al., 2020).

### 6.1 Key mutations and variants

During the early stages of the pandemic, SARS-CoV-2 S protein contained the aspartic acid (D) at position 614. As the pandemic progressed, however, a variant containing glycine at position 614 emerged and rapidly became dominant (Butowt et al., 2020; Boehm et al., 2021). One possible reason for the emergence of this mutation could be the furin cleavage site in the S protein of SARS-CoV-2 makes it more prone to S1 shedding, which reduces its infectivity compared to SARS-CoV. To compensate for this disadvantage, the SARS-CoV-2 S protein has seemingly developed a stronger intermolecular association between the S1 and S2 subunits through the D614G mutation (Jackson et al., 2022). Furthermore, the D614G mutation favors the open conformational state of the S protein, leading to increased RBD exposure and higher binding affinity to the ACE2 host receptor (Butowt et al., 2020). Regardless of its mechanism for enhanced infectivity, the widespread presence of the D614G mutation in most subsequent SARS-CoV-2 isolate suggests that it is indeed a beneficial mutation for adaptation to humans.

In addition to the D614G mutation, the SARS-CoV-2 variants Alpha (lineage B.1.1.7), Beta (B.1.351) and Gamma (P.1), which were first identified in the United Kingdom, South Africa and Brazil, respectively, carry a common N501Y mutation (Andreano et al., 2021; Faria et al., 2021; Harvey et al., 2021; The COVID-19 Genomics UK (COG-UK) consortium et al., 2021; Tian et al., 2022). The N501Y mutation substitutes asparagine with tyrosine at position 501, forms a potential aromatic ring-ring interaction and an additional hydrogen bond with ACE2, and reduces the polarity of critical residues in RBD, thereby increasing the affinity between RBD and the cellular surface of ACE2 (Tian et al., 2022). The Delta (B.1.617.2), first identified in India, does not carry the N501Y mutation. However, it possesses the P681R mutation in the furin cleavage site, which might contribute to increased transmissibility of the variant. Additionally, the Delta variant (B.1.617.2) also carries the L452R mutation, which causes moderate loss of susceptibility to neutralizing antibodies, allowing the variant to escape the human HLA-A24-presented T-cell response (Motozono et al., 2021). Many studies showed Delta (B.1.617.2) was associated with increased severity of illness and risk of death (Fisman and Tuite, 2021; Ong et al., 2022). There has been controversy regarding whether the Delta (B.1.617.2) variant exhibits higher viral loads in the upper respiratory tract. Some studies have suggested increased viral loads associated with the Delta variant (Costa et al., 2022; Li B. et al., 2022). However, in a cohort study conducted by Ong et al. (2022), they reported comparable SARS-CoV-2 Delta and Alpha RNA loads in nasopharyngeal specimens (NP) at the time of SARS-CoV-2 infection diagnosis using RT-PCR. Further research is needed to better understand the viral load dynamics and transmissibility of the Delta variant.

The COVID-19 pandemic enters a longer phase where immune escape will play a significant role in shaping the evolution of the S protein (Jackson et al., 2022). As the virus continues to circulate and encounters immune responses from vaccinated or previously infected individuals, selective pressure may drive the emergence of new variants with mutations that allow them to evade the immune system. E484K has been identified as an escape mutation that emerges during exposure to monoclonal antibodies C121 and C144 and convalescent plasma, and was the mutation described as able to reduce the neutralizing ability of a combination of monoclonal antibodies (REGN10989 and REGN10934) to an unmeasurable level in convalescent (Baum et al., 2020; Weisblum et al., 2020). The E484K mutation reduces binding of neutralizing antibodies and leads to partial immune escape, decreasing the efficacy of some antibody therapies or vaccines *in vitro* (Boehm et al., 2021). The combination of K417T + E484K + N501Y mutations may be more effective than any of these mutations alone in decreasing the neutralization of antibodies (Greaney et al., 2021). The SARS-CoV-2 variants Beta (B.1.351), Gamma (P.1) and Delta (B.1.617.2) have all experienced mutations at the E484 position.

The Omicron (B.1.1.529) variant, first identified in South Africa and Botswana, has attracted worldwide attention since its emergence owing to its high transmissibility and immune evasion capability (Fan et al., 2022; Shrestha et al., 2022). The Omicron variant did not develop from one of the earlier known variants, the phylogenetic analysis revealed that the Omicron variant formed a new monophyletic clade that is distant from other SARS-CoV-2 variants (Kandeel et al., 2022). There are three possible explanations for the development of the Omicron variant: silent evolution in a population with little sequencing, long-term evolution in one or a few individuals with chronic infection, or evolution in other animals (Fan et al., 2022; Shrestha et al., 2022). Some studies have suggested that the pre-outbreak mutations in Omicron might have accumulated in a non-human host, particularly rodents (Wei et al., 2021; Mallapaty, 2022; Smyth et al., 2022). The Omicron variant is not a single strain, but comprises three distinct sub-lineages: BA.1, BA.2, and BA.3. Subsequently, two other sub-lineages have also been identified, BA.4, BA.5 (Shrestha et al., 2022). The Omicron variant carries the highest number of mutations. In the BA.1 lineage, up to 60 mutations have been identified. Among these, as many as 38 mutations occur in the S protein, one in the envelope E protein, two in the membrane M protein, and six in the N protein. BA.2 lineage possesses 57 mutations, with 31 in the S protein (Fan et al., 2022; Kannan et al., 2022). Laboratory studies showed that the mutations in the Omicron variant altered the tropism of the virus. The Omicron tended to infect the upper respiratory tract, which can be attributed to that Omicron inefficiently utilize TMPRSS2 to enter cells, but mainly rely on the endocytic pathway, leading to a decrease in replication in the lung parenchyma and an enhanced ability to infect the upper respiratory tract (Hui et al., 2022; Meng et al., 2022). Increasing studies have indicated that infection with Omicron is associated with milder symptoms compared to earlier variants (Abdullah et al., 2022; Ulloa et al., 2022; Wolter et al., 2022). The Omicron variant exhibits a reduced capacity to induce syncytia formation in tissue culture. Syncytia formation has been associated with increased disease severity (Meng et al., 2022; Suzuki et al., 2022). However, all these data were obtained in *in vitro* or animal models, while it is likely that the reduced pathogenicity of the Omicron variant is due to the rising levels of cell-mediated immunity resulting from previous infections and vaccinations (Fan et al., 2022). The Omicron variant is capable of evading the humoral immune response established by vaccination or previous infection with other variants. Additionally, most therapeutic monoclonal antibodies are ineffective against the Omicron variant. These are attributed to multiple mutations and deletions in the S protein of the Omicron variant render a part of the S protein unrecognizable to the antibodies developed by natural infection or vaccination (Zhang and Holmes, 2020; Zhang et al., 2021; Iketani et al., 2022; VanBlargan et al., 2022). Fortunately, components of the cellular immune response, such as T cells, are still able to target Omicron and provide protection from severe outcomes. Data from La Jolla Institute has revealed that, despite several mutations in S protein, on average 94% of CD8 and 91% of CD4 epitopes of Omicron are still completely conserved, suggesting this variant has not evolved extensive T-cell escape mutations at this time (Shrestha et al., 2022).

The SARS-CoV-2 Omicron sub-variant BA.2.86 was first identified in Denmark in August 2023 (Rasmussen et al., 2023). Compared to its ancestor BA.2, the BA.2.86 sub-variant of Omicron carries 29 substitutions, 4 deletions, and 1 insertion in the S protein, suggesting an increased ability to evade antibodies induced by vaccination and previous infections (Looi, 2023; Mahase, 2023). The emergence of BA.2.86 highlights the continuous evolution of the SARS-CoV-2, influenced by global immunity levels, vaccination campaigns, and treatment strategies (Satapathy et al., 2024).

### 6.2 Quasispecies

Consensus genomic sequences of SARS-CoV-2 variants represent the genomes most frequently found in the clinical samples of patients, which have been widely used to monitor the global spread of the virus during the COVID-19 pandemic (Colson et al., 2023; Messali et al., 2023). However, these genomes represent the most frequently observed viral genomes and do not capture the full extent of their diversity (Colson et al., 2023). With advancements in sequencing techniques, studies have reported intrahost genetic diversity of SARS-CoV-2 in specific patient groups such as immunocompromised individuals, chronically infected patients, those treated with monoclonal antibodies targeting the spike protein, and within different body compartments of a single patient (Colson et al., 2023). This suggests that SARS-CoV-2 infections consist of a population of closely related viral variants, referred to as quasispecies (Colson et al., 2023; Messali et al., 2023).

A viral quasispecies is defined as the dynamic distribution of nonidentical but closely related mutants, variants, recombinant, or reassortant viral genomes (Colson et al., 2023; Messali et al., 2023; Delgado et al., 2024). This concept reflects the understanding that populations typically consist of distinct “types” characterized by certain criteria rather than fully identical individuals. Consequently, virus isolates or strains cannot be considered as linked to a single sequence but rather encompass a collection of sequences (Colson et al., 2023). It is currently hypothesized that hosts are infected by a diverse ensemble of viruses, termed the “wild bunch,” rather than multiple virions sharing the same genome. Each virion within this “wild bunch” may exhibit different virulence and tissue tropisms, enabling adaptation to various organs and potentially resulting in diseases of differing nature (Colson et al., 2023). The wide range of symptoms observed in different organs during the acute phase of SARS-CoV-2 infection aligns with the concept of the “wild bunch.” The presence of intrahost genomic variability leads to antigenic diversity and specific phenotypic characteristics, which influence replicative capacity, transmissibility, host range and tropism, susceptibility to immune responses, vaccines, and antivirals (Colson et al., 2023).

Quasispecies has revolutionized our understanding of viral evolution, particularly for RNA viruses. The concept highlights the dynamic and ever-changing nature of viral populations, leading to new insights in viral pathogenesis, vaccine development, and antiviral therapy.

## 7 Treatment

Current therapeutic strategies for SARS-CoV-2 infection include symptomatic treatment, supportive care and repurposed drugs. The main goal of these interventions is to inhibit the entry of the virus into cells. Extrinsic import of human recombinant soluble ACE2 (hrsACE2) can neutralize the virus in the serum (Zhang H. et al., 2020; Nazerian et al., 2021), and antibodies or small molecule drugs with higher binding affinity for the RBD of the virus may block viral attachment (Shang et al., 2020). However, the RBD is poorly conserved across the coronavirus family. Targeting membrane fusion, one of the most conserved regions of the S protein, has potential in treating broad-spectrum pan-CoV disease in the future (Xia et al., 2019; Walls et al., 2020). Xia et al. (2019) identified peptides that target other human coronaviruses with fusion inhibitory activity against membrane fusion domains, suggesting that the S2 protein subunit of SARS-CoV-2 could be a therapeutic target. Aptamers, consisting of single-stranded DNA or RNA molecules, possess the ability to bind to specific targets by adopting a unique 3D structure (Zhang et al., 2023). Currently, there is ongoing development of aptamers targeting the S protein of SARS-CoV-2. Studies have identified MSA52 as a universal aptamer capable of binding to the trimeric S proteins found in various SARS-CoV-2 variants of concern (Zhang et al., 2023). This broad-spectrum binding capability is highly significant for the development of effective antiviral treatments against the virus. As SARS-CoV-2 entry requires endosome acidification, neutralizing the acidic environment of endosomes may impair viral entry pathways (Wang et al., 2020; Nazerian et al., 2021). The antiviral peptide 8P9R was found to be able to cross-link viruses and inhibit endosomal acidification simultaneously to block viral entry (Zhao et al., 2021). Alternatively, TMPRSS2 and lysosomal cathepsins could be targets for preventing viral entry into cells (Hoffmann et al., 2020).

Conventional antivirals such as remdesivir, which inhibit viral RNA synthesis, have *in vitro* activity against SARS-CoV-2 (Wang et al., 2020). However, remdesivir needs to be administered intravenously, limiting its widespread use during the pandemic. The oral analog of remdesivir, VV116, has improved *in vitro* antiviral activity and selectivity with satisfactory safety, tolerability, and pharmacokinetic profiles in healthy subjects (Qian et al., 2022). A real-world study demonstrated that VV116 treatment resulted in a marked acceleration of viral shedding in patients infected with the SARS-CoV-2 omicron variant who received VV116 within 5 days after the first positive test (Shen et al., 2022). Compared to paxlovid, a new oral drug consisting of the second-generation protease inhibitor nirmatrelvir co-packaged with the pharmaceutical enhancer ritonavir that has been recommended for use in WHO guidelines, VV116 exhibited a similar time to sustained clinical recovery with fewer safety concerns in symptomatic adults with mild to moderate COVID-19 at risk of progression (Reina and Iglesias, 2022; Cao et al., 2023). Molnupiravir, an oral nucleoside analog with broad-spectrum antiviral activity and resilience to drug resistance, is an isopropyl ester prodrug of β-D-N4-hydroxycytidine (NHC; EIDD-1931) that targets the RdRp (Hadj Hassine et al., 2022). Molnupiravir exerts its antiviral effects through lethal mutagenesis, which increases G → A and C → U transition frequencies during replication to extinguish the virus (Kabinger et al., 2021; Hadj Hassine et al., 2022). It has shown promising efficacy and safety in Phase I/II/III clinical trials, but further studies are needed to evaluate its carcinogenic risks and genotoxicity (Hadj Hassine et al., 2022; Wen et al., 2022). Nanomaterials can be designed and manipulated to selectively target specific cells, reducing drug toxicity (Ao et al., 2023).

There is not enough evidence to support the use of corticosteroids in COVID-19 treatment. If glucocorticoids are used, they should not compromise the host defense mechanisms necessary to resist SARS-CoV-2 infection and must be used in combination with antiviral drugs (Bourgonje et al., 2020; van Eijk et al., 2021). Cytokine receptor antagonists such as anakinra and tocilizumab have been shown to reduce the inflammatory response (Cavalli and Dinarello, 2018; Berardicurti et al., 2020; Somers et al., 2020; Kim et al., 2021), while therapeutic plasma exchange (TPE) rapidly and non-selectively removes excess cytokines from the plasma, altering the proliferation status and function of lymphocytes and enhancing immunity against SARS-CoV-2 (Reeves and Winters, 2014; Knaup et al., 2018; Kim et al., 2021).

Anticoagulant therapy, specifically low molecular weight heparin (LMWH), has been proposed by the International Society of Thrombosis and Hemostasis (ISTH) for use in all hospitalized COVID-19 patients without contraindications (Bourgonje et al., 2020). However, some patients on prophylactic LMWH remain at risk of thrombotic complications due to potential heparin resistance caused by increased levels of NETs, therefore, higher therapeutic doses of LMWH would be required (Middleton et al., 2020). Inhibiting or catabolizing NETs may be a therapeutic avenue to explore, with neonatal NET inhibitory factor (nNIF) showing promise in decreasing NET formation induced by COVID-19 plasma *in vitro* (Middleton et al., 2020). Synthesized nNIF based on the endogenous sequence may have clinical applications (Middleton et al., 2020).

## 8 Vaccine

Vaccination is a crucial strategy in preventing SARS-CoV-2 infection or reducing disease severity. A major vaccine strategy was to induce antibodies that prevent the interaction between the RBD of SARS-CoV-2 and ACE2 (Pandey et al., 2021). Currently, over 150 vaccines are in various stages of development, including mRNA vaccines, adenoviral vector vaccines, inactivated vaccines and recombinant vaccines. Some of the most promising vaccine candidates include NVX-CoV2373, BNT162b2, mRNA-1273, Sputnik V, AZD1222, BBIBP-CorV, Covaxin, Ad26.CoV.S, and CoronaVac, with reported efficacy ranging from 51% to 96% (Hadj, 2022). The RBD of the S1 subunit contains major antigenic determinants, allowing the development of vaccines targeting the RBD (Du et al., 2009). Targeting the membrane fusion S2 subunit has been less successful due to its lower immunogenicity (Du et al., 2009; Shang et al., 2020).

SARS-CoV-2 variants have raised concerns about potential impact on vaccine efficacy (VE; Garcia-Beltran et al., 2021; Hadj, 2022). The VE of most vaccines is preserved against the alpha and beta variants, but reduced significantly against other variants (Harvey et al., 2021; Fiolet et al., 2022). mRNA vaccines, AZD1222 and CoronaVac are effective against the alpha, beta, gamma, and delta variants in preventing severe infections (Fiolet et al., 2022). mRNA vaccines may be beneficial for combating new viral strains due to the fact that they are capable of undergoing modifications through altering their sequence (Xu S. et al., 2020). Cellular immune responses induced by vaccination have shown strong cross-protection against variants despite their ability to evade humoral immunity (Moss, 2022). Mixing vaccines from different platforms has resulted in higher levels of neutralizing antibodies and stronger cellular immune responses, making it an attractive vaccination strategy (Rashedi et al., 2022). Table 1 provides detailed knowledge about the variants and their VE values for different vaccines.

**Table 1:** SARS-CoV-2 variants and vaccine efficacy.

New WHO name	Alpha	Beta	Gamma	Delta	Omicron
Strain name	B.1.1.7	B.1.351	P.1	B.1.617.2	B.1.1.529
Main spike mutations	69/70del, N501Y, A570D, D614G, P681H, T716I, S982A, D1118H (Harvey et al., 2021)	N501Y, E484K, K417N, D614G (Tian et al., 2022)	N501Y, K417T, E484K, D614G (Harvey et al., 2021)	D111D, G142D, L452R, E484R, E484Q, D614G, P681R (Harvey et al., 2021)	G339D, N440K, S477N, T478K, N501Y, K417N, G446S, E484A, Q493R, G496S, Q498R (Thakur and Ratho, 2022)
Vaccines efficacy against infection	NVX-CoV2373:85.6%–89.5% (Abu-Raddad et al., 2021; Harvey et al., 2021; Mahase, 2021)	NVX-CoV2373:60% (Mahase, 2021)	AZD1222:77.0% (Campos et al., 2021)	ChAdOx1nCoV-19:67% (Lopez Bernal et al., 2021)	BNT162b2:43%–86%
				mRNA-1273:76%–84%(Pouwels et al., 2021)	
	mRNA-1273:84%–100% (Chemaitelly et al., 2021; Seppälä et al., 2021)	AZD1222:10.4% (Madhi et al., 2021)	Ad26.COV2.S:68.1% (Sadoff et al., 2021)	BNT162b2:42%–79% (Tang et al., 2021)	mRNA-1273:58%–89%
	ChAdOx1nCoV-19:74.5% (Lopez Bernal et al., 2021)			AZD1222:60%–67%(Fiolet et al., 2022)	
	BNT162b2:78%–95% (Fiolet et al., 2022)	mRNA-1273:96.4% (Chemaitelly et al., 2021)		Sputnik V:90% (Hadj, 2022)	J&J:13%–86% (Thakur and Ratho, 2022)
	AZD1222:79% (Pouwels et al., 2021)	BNT162b2:72.1%–75% (Abu-Raddad et al., 2021; Zheng et al., 2022)		CoronaVac:59% (Li et al., 2021)	

Intramuscular (IM) injection of vaccines currently in use and under development fails to activate mucosal immunity, which can prevent virus infection through the upper respiratory tract (Alu et al., 2022). Intranasal (IN) administration of vaccines is a promising preventative strategy for SARS-CoV-2 due to induction of broad immune response-neutralizing IgG, mucosal IgA and T cell responses (Choudhary et al., 2021). IN vaccination results in robust mucosal and humoral immune responses, providing full protection of the respiratory tract and reducing virus concentrations in nasal swabs in animal models (Bricker et al., 2020; Hassan et al., 2020; van Doremalen et al., 2021). Single-dose IN immunization with chimpanzee Ad-vectored vaccine is superior to IM immunization in inducing tripartite protective immunity consisting of local and systemic antibody responses, mucosal tissue resident memory T cells, and mucosal trained innate immunity (Afkhami et al., 2022). I IN vaccines express additional conserved SARS-CoV-2 antigens to broaden T cell immunity, making them effective against both ancestral SARS-CoV-2 and its variants, compensating for the limitations of current IM vaccines (Afkhami et al., 2022; Chen et al., 2022).

Current vaccines exhibit reduced efficacy against the Omicron variant, primarily due to a reduction in the neutralizing activity of antibodies. Although routine doses of vaccination have shown limited efficacy in neutralizing the Omicron variant, substantial evidence has demonstrated that booster vaccinations are more effective in inducing neutralizing immunity against Omicron (Cameroni et al., 2022; Dejnirattisai et al., 2022; Planas et al., 2022). Several studies have shown that the administration of a booster shot can enhance vaccine effectiveness against the Omicron variant by 10–127 times (Gruell et al., 2022; Shrestha et al., 2022; Zhang et al., 2022). Therefore, booster vaccinations are necessary to provide effective protection against the Omicron variant. In addition, booster vaccination has been shown to enhance T-cell responses to Omicron S protein (GeurtsvanKessel et al., 2022; Naranbhai et al., 2022). Some researchers have developed specific vaccines targeting the Omicron variant by using RBD of Omicron. These Omicron-specific vaccines produced high titer of antibodies against Omicron itself in animal models, but few to none against other variants (Wu et al., 2022). These findings have raised interest in variant-specific vaccines and have spurred advancements in vaccine development.

## 9 Discussion

Although the global pandemic of SARS-CoV-2 is fading, populations affected by the virus continue to increase, and a continued focus on it is necessary. The complex pathogenesis of SARS-CoV-2 requires multilevel approaches to tackle the COVID-19 pandemic, with controlling the inflammatory response being as important as targeting the virus. A two-step therapeutic strategy has been proposed, consisting of antiviral drugs during the primary phase of the disease to reduce viral load and assist the immune system in combating the virus and immunomodulatory therapy during the secondary phase when protective effects on the immune system are no longer prominent, and unrestrained activity leads to cytokine storms.

Current therapeutic strategies for COVID-19 are mainly supportive care and repurposed drugs, which are not specific to SARS-CoV-2 and may have off-target effects (Nazerian et al., 2021). Researchers are developing specific agents to target the interaction of SARS-CoV-2 RBD with ACE2 and optimize the combination of antiviral agents and immunotherapeutic approaches. Targeting membrane fusion is attractive since the RBD is poorly conserved, and this approach may benefit future broad-spectrum pan-CoV disease treatment. Further studies on host immune response to SARS-CoV-2 are necessary to identify critical cytokines that contribute to disease progression, which can aid in designing timely therapeutic interventions to prevent severe outcomes. The emergence of BA.2.86 confirms the unpredictable nature of the COVID-19 pandemic, emphasizing the need for ongoing research, adaptability, and global collaboration. The emergence of new mutated variants compromises current vaccination strategies’ effectiveness, but booster vaccination, mixing vaccines and administering vaccines via the IN route to establish mucosal immunity may improve vaccine effectiveness. Specific vaccines targeting certain variants are also receiving growing attention as a strategy to combat various variants.

## Author contributions

XL: Writing – original draft, Writing – review & editing. ZM: Writing – review & editing. ZL: Writing – review & editing. PR: Writing – review & editing.

## Footnotes

^1^https://covid19.WHO.int./

## References

[ref1] Abdullah F. Myers J. Basu D. Tintinger G. Ueckermann V. Mathebula M.. (2022). Decreased severity of disease during the first global omicron variant covid-19 outbreak in a large hospital in tshwane, South Africa. Int. J. Infect. Dis. 116, 38–42. doi: 10.1016/j.ijid.2021.12.357, PMID: 34971823 PMC8713416

[ref2] Abu-Raddad L. J. Chemaitelly H. Butt A. A. National Study Group for COVID-19 Vaccination (2021). Effectiveness of the BNT162b2 Covid-19 vaccine against the B.1.1.7 and B.1.351 variants. N. Engl. J. Med. 385, 187–189. doi: 10.1056/NEJMc2104974, PMID: 33951357 PMC8117967

[ref3] Ackermann M. Verleden S. E. Kuehnel M. Haverich A. Welte T. Laenger F.. (2020). Pulmonary vascular Endothelialitis, thrombosis, and angiogenesis in Covid-19. N. Engl. J. Med. 383, 120–128. doi: 10.1056/NEJMoa2015432, PMID: 32437596 PMC7412750

[ref4] Afkhami S. D’Agostino M. R. Zhang A. Stacey H. D. Marzok A. Kang A.. (2022). Respiratory mucosal delivery of next-generation COVID-19 vaccine provides robust protection against both ancestral and variant strains of SARS-CoV-2. Cell 185, 896–915. doi: 10.1016/j.cell.2022.02.005, PMID: 35180381 PMC8825346

[ref5] Alonso-Canovas A. Kurtis M. M. Gomez-Mayordomo V. Macías-García D. Gutiérrez Viedma Á. Mondragón Rezola E.. (2023). Functional neurological disorders after COVID-19 and SARS-CoV-2 vaccines: a national multicentre observational study. J. Neurol. Neurosurg. Psychiatry 94, 776–777. doi: 10.1136/jnnp-2022-330885, PMID: 36889911

[ref6] Alu A. Chen L. Lei H. Wei Y. Tian X. Wei X. (2022). Intranasal COVID-19 vaccines: from bench to bed. EBioMedicine 76:103841. doi: 10.1016/j.ebiom.2022.103841, PMID: 35085851 PMC8785603

[ref7] Andreano E. Piccini G. Licastro D. Casalino L. Johnson N. V. Paciello I.. (2021). SARS-CoV-2 escape from a highly neutralizing COVID-19 convalescent plasma. Proc. Natl. Acad. Sci. U. S. A. 118:54118. doi: 10.1073/pnas.2103154118 PMC8433494 34417349

[ref8] Ao D. He X. Liu J. Xu L. (2023). Strategies for the development and approval of COVID-19 vaccines and therapeutics in the post-pandemic period. Signal Transduct. Target. Ther. 8:466. doi: 10.1038/s41392-023-01724-w, PMID: 38129394 PMC10739883

[ref9] Arvin A. M. Fink K. Schmid M. A. Cathcart A. Spreafico R. Havenar-Daughton C.. (2020). A perspective on potential antibody-dependent enhancement of SARS-CoV-2. Nature 584, 353–363. doi: 10.1038/s41586-020-2538-8, PMID: 32659783

[ref10] Baum A. Fulton B. O. Wloga E. Copin R. Pascal K. E. Russo V.. (2020). Antibody cocktail to SARS-CoV-2 spike protein prevents rapid mutational escape seen with individual antibodies. Science 369, 1014–1018. doi: 10.1126/science.abd0831, PMID: 32540904 PMC7299283

[ref11] Berardicurti O. Ruscitti P. Ursini F. D'Andrea S. Ciaffi J. Meliconi R.. (2020). Mortality in tocilizumab-treated patients with COVID-19: a systematic review and meta-analysis. Clin. Exp. Rheumatol. 38, 1247–1254. PMID: 33275094

[ref12] Blanco-Melo D. Nilsson-Payant B. E. Liu W. C. Uhl S. Hoagland D. Møller R.. (2020). Imbalanced host response to SARS-CoV-2 drives development of COVID-19. Cell 181, 1036–1045. doi: 10.1016/j.cell.2020.04.026, PMID: 32416070 PMC7227586

[ref13] Boehm E. Kronig I. Neher R. A. Eckerle I. Vetter P. Kaiser L.. (2021). Novel SARS-CoV-2 variants: the pandemics within the pandemic. Clin. Microbiol. Infect. 27, 1109–1117. doi: 10.1016/j.cmi.2021.05.022, PMID: 34015535 PMC8127517

[ref14] Borczuk A. C. (2021). Pulmonary pathology of COVID-19: a review of autopsy studies. Curr. Opin. Pulm. Med. 27, 184–192. doi: 10.1097/MCP.0000000000000761 33399353

[ref15] Bourgonje A. R. Abdulle A. E. Timens W. Hillebrands J. L. Navis G. J. Gordijn S. J.. (2020). Angiotensin-converting enzyme 2 (ACE2), SARS-CoV-2 and the pathophysiology of coronavirus disease 2019 (COVID-19). J. Pathol. 251, 228–248. doi: 10.1002/path.5471, PMID: 32418199 PMC7276767

[ref16] Bournazos S. Gupta A. Ravetch J. V. (2020). The role of IgG fc receptors in antibody-dependent enhancement. Nat. Rev. Immunol. 20, 633–643. doi: 10.1038/s41577-020-00410-0, PMID: 32782358 PMC7418887

[ref17] Bricker T. L. Darling T. L. Hassan A. O. Harastani H. H. Soung A. Jiang X.. (2020). A single intranasal or intramuscular immunization with chimpanzee adenovirus vectored SARS-CoV-2 vaccine protects against pneumonia in hamsters. bioRxiv [Preprint]. 10.1016/j.celrep.2021.109400 PMC8238649 34245672

[ref18] Brinkmann V. Reichard U. Goosmann C. Fauler B. Uhlemann Y. Weiss D. S.. (2004). Neutrophil extracellular traps kill bacteria. Science 303, 1532–1535. doi: 10.1126/science.1092385 15001782

[ref19] Butowt R. Bilinska K. Von Bartheld C. S. (2020). Chemosensory dysfunction in COVID-19: integration of genetic and epidemiological data points to D614G spike protein variant as a contributing factor. ACS Chem. Nerosci. 11, 3180–3184. doi: 10.1021/acschemneuro.0c00596, PMID: 32997488 PMC7581292

[ref20] Cameroni E. Bowen J. E. Rosen L. E. Saliba C. Zepeda S. K. Culap K.. (2022). Broadly neutralizing antibodies overcome SARS-CoV-2 omicron antigenic shift. Nature 602, 664–670. doi: 10.1038/s41586-021-04386-2, PMID: 35016195 PMC9531318

[ref21] Campos K. R. Sacchi C. T. Abbud A. Caterino-de-Araujo A. (2021). SARS-CoV-2 variants in severely symptomatic and deceased persons who had been vaccinated against COVID-19 in São Paulo, Brazil. Rev. Panam. Salud Publica 45:e126. doi: 10.26633/RPSP.2021.126 34707647 PMC8544615

[ref22] Cao Z. Gao W. Bao H. Feng H. Mei S. Chen P.. (2023). VV116 versus Nirmatrelvir-ritonavir for Oral treatment of Covid-19. N. Engl. J. Med. 388, 406–417. doi: 10.1056/NEJMoa2208822, PMID: 36577095 PMC9812289

[ref23] Cavalli G. Dinarello C. A. (2018). Anakinra therapy for non-cancer inflammatory diseases. Front. Pharmacol. 9:1157. doi: 10.3389/fphar.2018.01157, PMID: 30459597 PMC6232613

[ref24] Chemaitelly H. Yassine H. M. Benslimane F. M. al Khatib H. A. Tang P. Hasan M. R.. (2021). mRNA-1273 COVID-19 vaccine effectiveness against the B.1.1.7 and B.1.351 variants and severe COVID-19 disease in Qatar. Nat. Med. 27, 1614–1621. doi: 10.1038/s41591-021-01446-y, PMID: 34244681

[ref25] Chen J. Wang P. Yuan L. Zhang L. Zhang L. Zhao H.. (2022). A live attenuated virus-based intranasal COVID-19 vaccine provides rapid, prolonged, and broad protection against SARS-CoV-2. Sci. Bull. 67, 1372–1387. doi: 10.1016/j.scib.2022.05.018, PMID: 35637645 PMC9134758

[ref26] Choudhary O. P. Priyanka M. T. A. Mohammed T. A. Singh I. (2021). Intranasal COVID-19 vaccines: is it a boon or bane? Int. J. Surg. 94:106119. doi: 10.1016/j.ijsu.2021.106119, PMID: 34536600 PMC8443315

[ref27] Chu H. Chan J. F. Wang Y. Yuen T. T. Chai Y. Hou Y.. (2020). Comparative replication and immune activation profiles of SARS-CoV-2 and SARS-CoV in human lungs: an ex vivo study with implications for the pathogenesis of COVID-19. Clin. Infect. Dis. 71, 1400–1409. doi: 10.1093/cid/ciaa410, PMID: 32270184 PMC7184390

[ref28] Colson P. Bader W. Fantini J. Dudouet P. Levasseur A. Pontarotti P.. (2023). From viral democratic genomes to viral wild bunch of quasispecies. J. Med. Virol. 95:e29209. doi: 10.1002/jmv.29209, PMID: 37937701

[ref29] Costa R. Olea B. Bracho M. A. Albert E. de Michelena P. Martínez-Costa C.. (2022). RNA viral loads of SARS-CoV-2 alpha and Delta variants in nasopharyngeal specimens at diagnosis stratified by age, clinical presentation and vaccination status. J. Infect. 84, 579–613. doi: 10.1016/j.jinf.2021.12.018, PMID: 34953901 PMC8694784

[ref30] das S. Hensley S. E. David A. Schmidt L. Gibbs J. S. Puigbò P.. (2011). Fitness costs limit influenza a virus hemagglutinin glycosylation as an immune evasion strategy. Proc. Natl. Acad. Sci. U. S. A. 108, E1417–E1422. doi: 10.1073/pnas.1108754108, PMID: 22106257 PMC3251056

[ref31] Davies N. G. Abbott S. Barnard R. C. Jarvis C. I. Kucharski A. J. Munday J. D.. (2021). Estimated transmissibility and impact of SARS-CoV-2 lineage B.1.1.7 in England. Science 372:3055. doi: 10.1126/science.abg3055, PMID: 33658326 PMC8128288

[ref32] Dazert E. Neumann-Haefelin C. Bressanelli S. Fitzmaurice K. Kort J. Timm J.. (2009). Loss of viral fitness and cross-recognition by CD8+ T cells limit HCV escape from a protective HLA-B27-restricted human immune response. J. Clin. Invest. 119, 376–386. doi: 10.1172/JCI36587, PMID: 19139562 PMC2631298

[ref33] de Carvalho Santuchi M. Dutra M. F. Vago J. P. Lima K. M. Galvão I. de Souza-Neto F. P.. (2019). Angiotensin-(1-7) and Alamandine promote anti-inflammatory response in macrophages in vitro and in vivo. Mediators Inflamm. 2019, 1–14. doi: 10.1155/2019/2401081 PMC6409041 30918468

[ref34] de Melo G. D. Lazarini F. Levallois S. Hautefort C. Michel V. Larrous F.. (2021). COVID-19-related anosmia is associated with viral persistence and inflammation in human olfactory epithelium and brain infection in hamsters. Sci. Transl. Med. 13:8396. doi: 10.1126/scitranslmed.abf8396 PMC8158965 33941622

[ref35] de Wit E. van Doremalen N. Falzarano D. Munster V. J. (2016). SARS and MERS: recent insights into emerging coronaviruses. Nat. Rev. Microbiol. 14, 523–534. doi: 10.1038/nrmicro.2016.81, PMID: 27344959 PMC7097822

[ref36] Dejnirattisai W. Huo J. Zhou D. Zahradník J. Supasa P. Liu C.. (2022). SARS-CoV-2 omicron-B.1.1.529 leads to widespread escape from neutralizing antibody responses. Cell 185, 467–484. doi: 10.1016/j.cell.2021.12.046, PMID: 35081335 PMC8723827

[ref37] Delgado S. Somovilla P. Ferrer-Orta C. Martínez-González B. Vázquez-Monteagudo S. Muñoz-Flores J.. (2024). Incipient functional SARS-CoV-2 diversification identified through neural network haplotype maps. Proc. Natl. Acad. Sci. U. S. A. 121:e2317851121. doi: 10.1073/pnas.2317851121, PMID: 38416684 PMC10927536

[ref38] Domizio J. D. Gulen M. F. Saidoune F. Thacker V. V. Yatim A. Sharma K.. (2022). The cGAS-STING pathway drives type I IFN immunopathology in COVID-19. Nature 603, 145–151. doi: 10.1038/s41586-022-04421-w, PMID: 35045565 PMC8891013

[ref39] Du L. He Y. Zhou Y. Liu S. Zheng B. J. Jiang S. (2009). The spike protein of SARS-CoV--a target for vaccine and therapeutic development. Nat. Rev. Microbiol. 7, 226–236. doi: 10.1038/nrmicro2090, PMID: 19198616 PMC2750777

[ref40] Ellul M. A. Benjamin L. Singh B. Lant S. Michael B. D. Easton A.. (2020). Neurological associations of COVID-19. Lancet Neurol. 19, 767–783. doi: 10.1016/S1474-4422(20)30221-0, PMID: 32622375 PMC7332267

[ref41] Fan Y. Li X. Zhang L. Wan S. Zhang L. Zhou F. (2022). SARS-CoV-2 omicron variant: recent progress and future perspectives. Signal Transduct. Target. Ther. 7:141. doi: 10.1038/s41392-022-00997-x, PMID: 35484110 PMC9047469

[ref42] Faria N. R. Mellan T. A. Whittaker C. Claro I. M. Candido D. D. S. Mishra S.. (2021). Genomics and epidemiology of the P.1 SARS-CoV-2 lineage in Manaus, Brazil. Science 372, 815–821. doi: 10.1126/science.abh2644, PMID: 33853970 PMC8139423

[ref43] Fernández-Castañeda A. Lu P. Geraghty A. C. Song E. Lee M. H. Wood J.. (2022). Mild respiratory COVID can cause multi-lineage neural cell and myelin dysregulation. Cell 185, 2452–2468. doi: 10.1016/j.cell.2022.06.008, PMID: 35768006 PMC9189143

[ref44] Fiolet T. Kherabi Y. MacDonald C. J. Ghosn J. Peiffer-Smadja N. (2022). Comparing COVID-19 vaccines for their characteristics, efficacy and effectiveness against SARS-CoV-2 and variants of concern: a narrative review. Clin. Microbiol. Infect. 28, 202–221. doi: 10.1016/j.cmi.2021.10.005, PMID: 34715347 PMC8548286

[ref45] Fisman D. N. Tuite A. R. (2021). Evaluation of the relative virulence of novel SARS-CoV-2 variants: a retrospective cohort study in Ontario, Canada. J Assoc Med Can 193, E1619–E1625. doi: 10.1503/cmaj.211248, PMID: 34610919 PMC8562985

[ref46] Forchette L. Sebastian W. Liu T. (2021). A comprehensive review of COVID-19 virology, vaccines, variants, and therapeutics. Curr Med Sci. 41, 1037–1051. doi: 10.1007/s11596-021-2395-1, PMID: 34241776 PMC8267225

[ref47] Gao Y. D. Ding M. Dong X. Zhang J. J. Kursat Azkur A. Azkur D.. (2021). Risk factors for severe and critically ill COVID-19 patients: a review. Allergy 76, 428–455. doi: 10.1111/all.14657 33185910

[ref48] Garcia-Beltran W. F. Lam E. C. St. Denis K. Nitido A. D. Garcia Z. H. Hauser B. M.. (2021). Multiple SARS-CoV-2 variants escape neutralization by vaccine-induced humoral immunity. Cell 184, 2372–2383. doi: 10.1016/j.cell.2021.03.013, PMID: 33743213 PMC7953441

[ref49] Georg P. Astaburuaga-García R. Bonaguro L. Brumhard S. Michalick L. Lippert L. J.. (2022). Complement activation induces excessive T cell cytotoxicity in severe COVID-19. Cell 185, 493–512. doi: 10.1016/j.cell.2021.12.040, PMID: 35032429 PMC8712270

[ref50] GeurtsvanKessel C. H. Geers D. Schmitz K. S. Mykytyn A. Z. Lamers M. M. Bogers S.. (2022). Divergent SARS-CoV-2 omicron-reactive T and B cell responses in COVID-19 vaccine recipients. Sci Immunol. 7:2202. doi: 10.1126/sciimmunol.abo2202 PMC8939771 35113647

[ref51] Greaney A. J. Loes A. N. Crawford K. H. D. Starr T. N. Malone K. D. Chu H. Y.. (2021). Comprehensive mapping of mutations in the SARS-CoV-2 receptor-binding domain that affect recognition by polyclonal human plasma antibodies. Cell Host Microbe 29, 463–476. doi: 10.1016/j.chom.2021.02.003, PMID: 33592168 PMC7869748

[ref52] Greene C. Connolly R. Brennan D. Laffan A. O’Keeffe E. Zaporojan L.. (2024). Blood-brain barrier disruption and sustained systemic inflammation in individuals with long COVID-associated cognitive impairment. Nat. Neurosci. 27, 421–432. doi: 10.1038/s41593-024-01576-9, PMID: 38388736 PMC10917679

[ref53] Gruell H. Vanshylla K. Tober-Lau P. Hillus D. Schommers P. Lehmann C.. (2022). mRNA booster immunization elicits potent neutralizing serum activity against the SARS-CoV-2 omicron variant. Nat. Med. 28, 477–480. doi: 10.1038/s41591-021-01676-0, PMID: 35046572 PMC8767537

[ref54] Gupta A. Madhavan M. V. Sehgal K. Nair N. Mahajan S. Sehrawat T. S.. (2020). Extrapulmonary manifestations of COVID-19. Nat. Med. 26, 1017–1032. doi: 10.1038/s41591-020-0968-3 32651579 PMC11972613

[ref55] Hadj H. I. (2022). Covid-19 vaccines and variants of concern: a review. Rev. Med. Virol. 32:e2313. doi: 10.1002/rmv.2313, PMID: 34755408 PMC8646685

[ref56] Hadj Hassine I. Ben M’hadheb M. Menéndez-Arias L. (2022). Lethal mutagenesis of RNA viruses and approved drugs with antiviral mutagenic activity. Viruses 14:841. doi: 10.3390/v14040841, PMID: 35458571 PMC9024455

[ref57] Hadjadj J. Yatim N. Barnabei L. Corneau A. Boussier J. Smith N.. (2020). Impaired type I interferon activity and inflammatory responses in severe COVID-19 patients. Science 369, 718–724. doi: 10.1126/science.abc6027, PMID: 32661059 PMC7402632

[ref58] Harvey W. T. Carabelli A. M. Jackson B. Gupta R. K. Thomson E. C. Harrison E. M.. (2021). SARS-CoV-2 variants, spike mutations and immune escape. Nat. Rev. Microbiol. 19, 409–424. doi: 10.1038/s41579-021-00573-0, PMID: 34075212 PMC8167834

[ref59] Hashimoto T. Perlot T. Rehman A. Trichereau J. Ishiguro H. Paolino M.. (2012). ACE2 links amino acid malnutrition to microbial ecology and intestinal inflammation. Nature 487, 477–481. doi: 10.1038/nature11228, PMID: 22837003 PMC7095315

[ref60] Hassan A. O. Kafai N. M. Dmitriev I. P. Fox J. M. Smith B. K. Harvey I. B.. (2020). A single-dose intranasal ChAd vaccine protects upper and lower respiratory tracts against SARS-CoV-2. Cell 183, 169–184. doi: 10.1016/j.cell.2020.08.026, PMID: 32931734 PMC7437481

[ref61] Heurich A. Hofmann-Winkler H. Gierer S. Liepold T. Jahn O. Pöhlmann S. (2014). TMPRSS2 and ADAM17 cleave ACE2 differentially and only proteolysis by TMPRSS2 augments entry driven by the severe acute respiratory syndrome coronavirus spike protein. J. Virol. 88, 1293–1307. doi: 10.1128/JVI.02202-13, PMID: 24227843 PMC3911672

[ref62] Ho C. Y. Salimian M. Hegert J. O’Brien J. Choi S. G. Ames H.. (2022). Postmortem assessment of olfactory tissue degeneration and microvasculopathy in patients with COVID-19. JAMA Neurol. 79, 544–553. doi: 10.1001/jamaneurol.2022.0154, PMID: 35404378 PMC9002725

[ref63] Hoffmann M. Kleine-Weber H. Schroeder S. Krüger N. Herrler T. Erichsen S.. (2020). SARS-CoV-2 cell entry depends on ACE2 and TMPRSS2 and is blocked by a clinically proven protease inhibitor. Cell 181, 271–280. doi: 10.1016/j.cell.2020.02.052, PMID: 32142651 PMC7102627

[ref64] Holter J. C. Pischke S. E. de Boer E. Lind A. Jenum S. Holten A. R.. (2020). Systemic complement activation is associated with respiratory failure in COVID-19 hospitalized patients. Proc. Natl. Acad. Sci. U. S. A. 117, 25018–25025. doi: 10.1073/pnas.2010540117, PMID: 32943538 PMC7547220

[ref65] Huang C. Wang Y. Li X. Ren L. Zhao J. Hu Y.. (2020). Clinical features of patients infected with 2019 novel coronavirus in Wuhan. Lancet 395, 497–506. doi: 10.1016/S0140-6736(20)30183-5, PMID: 31986264 PMC7159299

[ref66] Hue S. Beldi-Ferchiou A. Bendib I. Surenaud M. Fourati S. Frapard T.. (2020). Uncontrolled innate and impaired adaptive immune responses in patients with COVID-19 acute respiratory distress syndrome. Am. J. Respir. Crit. Care Med. 202, 1509–1519. doi: 10.1164/rccm.202005-1885OC, PMID: 32866033 PMC7706149

[ref67] Hui K. P. Y. Ho J. C. W. Cheung M. C. Ng K. C. Ching R. H. H. Lai K. L.. (2022). SARS-CoV-2 omicron variant replication in human bronchus and lung ex vivo. Nature 603, 715–720. doi: 10.1038/s41586-022-04479-6, PMID: 35104836

[ref68] Hurst K. R. Koetzner C. A. Masters P. S. (2009). Identification of in vivo-interacting domains of the murine coronavirus nucleocapsid protein. J. Virol. 83, 7221–7234. doi: 10.1128/JVI.00440-09, PMID: 19420077 PMC2704785

[ref69] Iketani S. Liu L. Guo Y. Liu L. Chan J. F. Huang Y.. (2022). Antibody evasion properties of SARS-CoV-2 omicron sublineages. Nature 604, 553–556. doi: 10.1038/s41586-022-04594-4, PMID: 35240676 PMC9021018

[ref70] Jackson C. B. Farzan M. Chen B. Choe H. (2022). Mechanisms of SARS-CoV-2 entry into cells. Nat. Rev. Mol. Cell Biol. 23, 3–20. doi: 10.1038/s41580-021-00418-x, PMID: 34611326 PMC8491763

[ref71] Jose R. J. Manuel A. (2020). Does coronavirus disease 2019 disprove the obesity paradox in acute respiratory distress syndrome? Obesity (Silver Spring) 28:1007. doi: 10.1002/oby.22835, PMID: 32294322 PMC7262201

[ref72] Kabinger F. Stiller C. Schmitzová J. Dienemann C. Kokic G. Hillen H. S.. (2021). Mechanism of molnupiravir-induced SARS-CoV-2 mutagenesis. Nat. Struct. Mol. Biol. 28, 740–746. doi: 10.1038/s41594-021-00651-0, PMID: 34381216 PMC8437801

[ref73] Kalfaoglu B. Almeida-Santos J. Tye C. A. Satou Y. Ono M. (2020). T-cell hyperactivation and paralysis in severe COVID-19 infection revealed by single-cell analysis. Front. Immunol. 11:589380. doi: 10.3389/fimmu.2020.589380, PMID: 33178221 PMC7596772

[ref74] Kandeel M. Mohamed M. E. M. Abd El-Lateef H. M. Venugopala K. N. El-Beltagi H. S. (2022). Omicron variant genome evolution and phylogenetics. J. Med. Virol. 94, 1627–1632. doi: 10.1002/jmv.27515, PMID: 34888894 PMC9015349

[ref75] Kannan S. R. Spratt A. N. Sharma K. Chand H. S. Byrareddy S. N. Singh K. (2022). Omicron SARS-CoV-2 variant: unique features and their impact on pre-existing antibodies. J. Autoimmun. 126:102779. doi: 10.1016/j.jaut.2021.102779, PMID: 34915422 PMC8666303

[ref76] Kawai T. Akira S. (2011). Toll-like receptors and their crosstalk with other innate receptors in infection and immunity. Immunity 34, 637–650. doi: 10.1016/j.immuni.2011.05.006, PMID: 21616434

[ref77] Khan M. Yoo S. J. Clijsters M. Backaert W. Vanstapel A. Speleman K.. (2021). Visualizing in deceased COVID-19 patients how SARS-CoV-2 attacks the respiratory and olfactory mucosae but spares the olfactory bulb. Cell 184, 5932–5949. doi: 10.1016/j.cell.2021.10.027 34798069 PMC8564600

[ref78] Kim J. S. Lee J. Y. Yang J. W. Lee K. H. Effenberger M. Szpirt W.. (2021). Immunopathogenesis and treatment of cytokine storm in COVID-19. Theranostics 11, 316–329. doi: 10.7150/thno.49713, PMID: 33391477 PMC7681075

[ref79] Knaup H. Stahl K. Schmidt B. M. W. Idowu TO Busch M. Wiesner O.. (2018). Early therapeutic plasma exchange in septic shock: a prospective open-label nonrandomized pilot study focusing on safety, hemodynamics, vascular barrier function, and biologic markers. Crit. Care 22:285. doi: 10.1186/s13054-018-2220-9, PMID: 30373638 PMC6206942

[ref80] Konno Y. Kimura I. Uriu K. Fukushi M. Irie T. Koyanagi Y.. (2020). SARS-CoV-2 ORF3b is a potent interferon antagonist whose activity is increased by a naturally occurring elongation variant. Cell Rep. 32:108185. doi: 10.1016/j.celrep.2020.108185, PMID: 32941788 PMC7473339

[ref81] Kuba K. Imai Y. Rao S. Gao H. Guo F. Guan B.. (2005). A crucial role of angiotensin converting enzyme 2 (ACE2) in SARS coronavirus-induced lung injury. Nat. Med. 11, 875–879. doi: 10.1038/nm1267, PMID: 16007097 PMC7095783

[ref82] Kumar S. Tao Q. Weaver S. Sanderford M. Caraballo-Ortiz M. A. Sharma S.. (2021). An evolutionary portrait of the progenitor SARS-CoV-2 and its dominant offshoots in COVID-19 pandemic. Mol. Biol. Evol. 38, 3046–3059. doi: 10.1093/molbev/msab118, PMID: 33942847 PMC8135569

[ref83] Lee J. S. Park S. Jeong H. W. Ahn J. Y. Choi S. J. Lee H.. (2020). Immunophenotyping of COVID-19 and influenza highlights the role of type I interferons in development of severe COVID-19. Sci Immunol. 5:1554. doi: 10.1126/sciimmunol.abd1554, PMID: 32651212 PMC7402635

[ref84] Lee W. S. Wheatley A. K. Kent S. J. DeKosky B. J. (2020). Antibody-dependent enhancement and SARS-CoV-2 vaccines and therapies. Nat. Microbiol. 5, 1185–1191. doi: 10.1038/s41564-020-00789-5 32908214 PMC12103240

[ref85] Leisman D. E. Ronner L. Pinotti R. Taylor M. D. Sinha P. Calfee C. S.. (2020). Cytokine elevation in severe and critical COVID-19: a rapid systematic review, meta-analysis, and comparison with other inflammatory syndromes. Lancet Respir. Med. 8, 1233–1244. doi: 10.1016/S2213-2600(20)30404-5, PMID: 33075298 PMC7567529

[ref86] Leung J. M. Yang C. X. Tam A. Shaipanich T. Hackett T. L. Singhera G. K.. (2020). ACE-2 expression in the small airway epithelia of smokers and COPD patients: implications for COVID-19. Eur. Respir. J. 55:2000688. doi: 10.1183/13993003.00688-2020, PMID: 32269089 PMC7144263

[ref87] Li B. Deng A. Li K. Hu Y. Li Z. Shi Y.. (2022). Viral infection and transmission in a large, well-traced outbreak caused by the SARS-CoV-2 Delta variant. Nat. Commun. 13:460. doi: 10.1038/s41467-022-28089-y, PMID: 35075154 PMC8786931

[ref88] Li X. Hou P. Ma W. Wang X. Wang H. Yu Z.. (2022). SARS-CoV-2 ORF10 suppresses the antiviral innate immune response by degrading MAVS through mitophagy. Cell. Mol. Immunol. 19, 67–78. doi: 10.1038/s41423-021-00807-4, PMID: 34845370 PMC8628139

[ref89] Li X. N. Huang Y. Wang W. Jing Q. L. Zhang C. H. Qin P. Z.. (2021). Effectiveness of inactivated SARS-CoV-2 vaccines against the Delta variant infection in Guangzhou: a test-negative case-control real-world study. Emerg Microbes Infect. 10, 1751–1759. doi: 10.1080/22221751.2021.1969291, PMID: 34396940 PMC8425710

[ref90] Liang Y. Wang M. L. Chien C. S. Yarmishyn A. A. Yang Y. P. Lai W. Y.. (2020). Highlight of immune pathogenic response and Hematopathologic effect in SARS-CoV, MERS-CoV, and SARS-Cov-2 infection. Front. Immunol. 11:1022. doi: 10.3389/fimmu.2020.01022, PMID: 32574260 PMC7236801

[ref91] Liu Q. Shi Y. Cai J. Duan Y. Wang R. Zhang H.. (2020). Pathological changes in the lungs and lymphatic organs of 12 COVID-19 autopsy cases. Natl. Sci. Rev. 7, 1868–1878. doi: 10.1093/nsr/nwaa247, PMID: 34676085 PMC7543449

[ref92] Looi M. K. (2023). Covid-19: scientists sound alarm over new BA.2.86 "Pirola" variant. BMJ 382:1964. doi: 10.1136/bmj.p1964 37620014

[ref93] Lopez Bernal J. Andrews N. Gower C. Gallagher E. Simmons R. Thelwall S.. (2021). Effectiveness of Covid-19 vaccines against the B.1.617.2 (Delta) variant. N Engl J Med Overseas Ed 385, 585–594. doi: 10.1056/NEJMoa2108891, PMID: 34289274 PMC8314739

[ref94] Madhi S. A. Baillie V. Cutland C. L. Voysey M. Koen A. L. Fairlie L.. (2021). Efficacy of the ChAdOx1 nCoV-19 Covid-19 vaccine against the B.1.351 variant. N. Engl. J. Med. 384, 1885–1898. doi: 10.1056/NEJMoa2102214, PMID: 33725432 PMC7993410

[ref95] Magalhaes G. S. Rodrigues-Machado M. D. G. Motta-Santos D. Campagnole-Santos M. J. Santos R. A. S. (2020). Activation of Ang-(1-7)/mas receptor is a possible strategy to treat coronavirus (SARS-CoV-2) infection. Front. Physiol. 11:730. doi: 10.3389/fphys.2020.00730, PMID: 32636762 PMC7318839

[ref96] Mahase E. (2021). Covid-19: Novavax vaccine efficacy is 86% against UK variant and 60% against South African variant. BMJ 372:n296. doi: 10.1136/bmj.n296 33526412

[ref97] Mahase E. (2023). Covid-19: new "Pirola" variant BA.2.86 continues to spread in UK and US. BMJ 382:2097. doi: 10.1136/bmj.p2097 37704230

[ref98] Mahmudpour M. Roozbeh J. Keshavarz M. Farrokhi S. Nabipour I. (2020). COVID-19 cytokine storm: the anger of inflammation. Cytokine 133:155151. doi: 10.1016/j.cyto.2020.155151, PMID: 32544563 PMC7260598

[ref99] Mallapaty S. (2022). Where did omicron come from? Three key theories. Nature 602, 26–28. doi: 10.1038/d41586-022-00215-2, PMID: 35091701

[ref100] Mao L. Jin H. Wang M. Hu Y. Chen S. He Q.. (2020). Neurologic manifestations of hospitalized patients with coronavirus disease 2019 in Wuhan, China. JAMA Neurol. 77, 683–690. doi: 10.1001/jamaneurol.2020.1127, PMID: 32275288 PMC7149362

[ref101] Mastellos D. C. Pires da Silva B. G. P. Fonseca B. A. L. Fonseca N. P. Auxiliadora-Martins M. Mastaglio S.. (2020). Complement C3 vs C5 inhibition in severe COVID-19: early clinical findings reveal differential biological efficacy. Clin. Immunol. 220:108598. doi: 10.1016/j.clim.2020.108598, PMID: 32961333 PMC7501834

[ref102] Matschke J. Lütgehetmann M. Hagel C. Sperhake J. P. Schröder A. S. Edler C.. (2020). Neuropathology of patients with COVID-19 in Germany: a post-mortem case series. Lancet Neurol. 19, 919–929. doi: 10.1016/S1474-4422(20)30308-2, PMID: 33031735 PMC7535629

[ref103] McBride R. van Zyl M. Fielding B. C. (2014). The coronavirus nucleocapsid is a multifunctional protein. Viruses 6, 2991–3018. doi: 10.3390/v6082991, PMID: 25105276 PMC4147684

[ref104] McGonagle D. O'Donnell J. S. Sharif K. Emery P. Bridgewood C. (2020b). Immune mechanisms of pulmonary intravascular coagulopathy in COVID-19 pneumonia. Lancet Rheumatol. 2, e437–e445. doi: 10.1016/S2665-9913(20)30121-1, PMID: 32835247 PMC7252093

[ref105] McGonagle D. Sharif K. O'Regan A. Bridgewood C. (2020a). The role of cytokines including Interleukin-6 in COVID-19 induced pneumonia and macrophage activation syndrome-like disease. Autoimmun. Rev. 19:102537. doi: 10.1016/j.autrev.2020.102537, PMID: 32251717 PMC7195002

[ref106] Meng B. Abdullahi A. Ferreira I. Goonawardane N. Saito A. Kimura I.. (2022). Altered TMPRSS2 usage by SARS-CoV-2 omicron impacts infectivity and fusogenicity. Nature 603, 706–714. doi: 10.1038/s41586-022-04474-x, PMID: 35104837 PMC8942856

[ref107] Messali S. Rondina A. Giovanetti M. Bonfanti C. Ciccozzi M. Caruso A.. (2023). Traceability of SARS-CoV-2 transmission through quasispecies analysis. J. Med. Virol. 95:e28848. doi: 10.1002/jmv.28848, PMID: 37294038

[ref108] Middleton E. A. He X. Y. Denorme F. Campbell R. A. Ng D. Salvatore S. P.. (2020). Neutrophil extracellular traps contribute to immunothrombosis in COVID-19 acute respiratory distress syndrome. Blood 136, 1169–1179. doi: 10.1182/blood.2020007008, PMID: 32597954 PMC7472714

[ref109] Minkoff J. M. tenOever B. (2023). Innate immune evasion strategies of SARS-CoV-2. Nat. Rev. Microbiol. 21, 178–194. doi: 10.1038/s41579-022-00839-1, PMID: 36631691 PMC9838430

[ref110] Miyakis S. Lockshin M. D. Atsumi T. Branch D. W. Brey R. L. Cervera R.. (2006). International consensus statement on an update of the classification criteria for definite antiphospholipid syndrome (APS). J. Thromb. Haemost. 4, 295–306. doi: 10.1111/j.1538-7836.2006.01753.x, PMID: 16420554

[ref111] Mortaz E. Tabarsi P. Varahram M. Folkerts G. Adcock I. M. (2020). The immune response and immunopathology of COVID-19. Front. Immunol. 11:2037. doi: 10.3389/fimmu.2020.02037, PMID: 32983152 PMC7479965

[ref112] Moss P. (2022). The T cell immune response against SARS-CoV-2. Nat. Immunol. 23, 186–193. doi: 10.1038/s41590-021-01122-w, PMID: 35105982

[ref113] Motozono C. Toyoda M. Zahradnik J. Saito A. Nasser H. Tan T. S.. (2021). SARS-CoV-2 spike L452R variant evades cellular immunity and increases infectivity. Cell Host Microbe 29, 1124–1136. doi: 10.1016/j.chom.2021.06.006, PMID: 34171266 PMC8205251

[ref114] Naranbhai V. Nathan A. Kaseke C. Berrios C. Khatri A. Choi S.. (2022). T cell reactivity to the SARS-CoV-2 omicron variant is preserved in most but not all individuals. Cell 185, 1041–1051. doi: 10.1016/j.cell.2022.01.029, PMID: 35202566 PMC8810349

[ref115] Nazerian Y. Vakili K. Ebrahimi A. Niknejad H. (2021). Developing cytokine storm-sensitive therapeutic strategy in COVID-19 using 8P9R chimeric peptide and soluble ACE2. Front. Cell Dev. Biol. 9:717587. doi: 10.3389/fcell.2021.717587, PMID: 34540833 PMC8446510

[ref116] Neuman B. W. Kiss G. Kunding A. H. Bhella D. Baksh M. F. Connelly S.. (2011). A structural analysis of M protein in coronavirus assembly and morphology. J. Struct. Biol. 174, 11–22. doi: 10.1016/j.jsb.2010.11.021, PMID: 21130884 PMC4486061

[ref117] Nieto-Torres J. L. DeDiego M. L. Verdiá-Báguena C. Jimenez-Guardeño J. M. Regla-Nava J. A. Fernandez-Delgado R.. (2014). Severe acute respiratory syndrome coronavirus envelope protein ion channel activity promotes virus fitness and pathogenesis. PLoS Pathog. 10:e1004077. doi: 10.1371/journal.ppat.1004077, PMID: 24788150 PMC4006877

[ref118] Noris M. Benigni A. Remuzzi G. (2020). The case of complement activation in COVID-19 multiorgan impact. Kidney Int. 98, 314–322. doi: 10.1016/j.kint.2020.05.013, PMID: 32461141 PMC7246017

[ref119] Ong S. W. X. Chiew C. J. Ang L. W. Mak T. M. Cui L. Toh M.. (2022). Clinical and virological features of severe acute respiratory syndrome coronavirus 2 (SARS-CoV-2) variants of concern: a retrospective cohort study comparing B.1.1.7 (alpha), B.1.351 (Beta), and B.1.617.2 (Delta). Clin. Infect. Dis. 75, e1128–e1136. doi: 10.1093/cid/ciab721 34423834 PMC8522361

[ref120] Pandey M. Ozberk V. Eskandari S. Shalash A. O. Joyce M. A. Saffran H. A.. (2021). Antibodies to neutralising epitopes synergistically block the interaction of the receptor-binding domain of SARS-CoV-2 to ACE 2. Clin Transl Immunol. 10:e1260. doi: 10.1002/cti2.1260, PMID: 33732459 PMC7937407

[ref121] Park A. Iwasaki A. (2020). Type I and type III interferons—induction, Signaling, evasion, and application to combat COVID-19. Cell Host Microbe 27, 870–878. doi: 10.1016/j.chom.2020.05.008, PMID: 32464097 PMC7255347

[ref122] Perico L. Benigni A. Casiraghi F. Ng L. F. P. Renia L. Remuzzi G. (2021). Immunity, endothelial injury and complement-induced coagulopathy in COVID-19. Nat. Rev. Nephrol. 17, 46–64. doi: 10.1038/s41581-020-00357-4, PMID: 33077917 PMC7570423

[ref123] Planas D. Saunders N. Maes P. Guivel-Benhassine F. Planchais C. Buchrieser J.. (2022). Considerable escape of SARS-CoV-2 omicron to antibody neutralization. Nature 602, 671–675. doi: 10.1038/s41586-021-04389-z, PMID: 35016199

[ref124] Pouwels K. B. Pritchard E. Matthews P. C. Stoesser N. Eyre D. W. Vihta K. D.. (2021). Effect of Delta variant on viral burden and vaccine effectiveness against new SARS-CoV-2 infections in the UK. Nat. Med. 27, 2127–2135. doi: 10.1038/s41591-021-01548-7, PMID: 34650248 PMC8674129

[ref125] Pyrc K. Dijkman R. Deng L. Jebbink M. F. Ross H. A. Berkhout B.. (2006). Mosaic structure of human coronavirus NL63, one thousand years of evolution. J. Mol. Biol. 364, 964–973. doi: 10.1016/j.jmb.2006.09.074, PMID: 17054987 PMC7094706

[ref126] Qian H. J. Wang Y. Zhang M. Q. Xie Y. C. Wu Q. Q. Liang L. Y.. (2022). Safety, tolerability, and pharmacokinetics of VV116, an oral nucleoside analog against SARS-CoV-2, in Chinese healthy subjects. Acta Pharmacol. Sin. 43, 3130–3138. doi: 10.1038/s41401-022-00895-6, PMID: 35296780 PMC8924727

[ref127] Rashedi R. Samieefar N. Masoumi N. Mohseni S. Rezaei N. (2022). COVID-19 vaccines mix-and-match: the concept, the efficacy and the doubts. J. Med. Virol. 94, 1294–1299. doi: 10.1002/jmv.27463, PMID: 34796525 PMC8661746

[ref128] Rasmussen M. Møller F. T. Gunalan V. Baig S. Bennedbæk M. Christiansen L. E.. (2023). First cases of SARS-CoV-2 BA.2.86 in Denmark, 2023. Euro Surveill. 28:460. doi: 10.2807/1560-7917.ES.2023.28.36.2300460, PMID: 37676147 PMC10486197

[ref129] Reeves H. M. Winters J. L. (2014). The mechanisms of action of plasma exchange. Br. J. Haematol. 164, 342–351. doi: 10.1111/bjh.12629 24172059

[ref130] Reina J. Iglesias C. (2022). Nirmatrelvir plus ritonavir (Paxlovid) a potent SARS-CoV-2 3CLpro protease inhibitor combination. Rev. Esp. Quimioter. 35, 236–240. doi: 10.37201/req/002.2022, PMID: 35183067 PMC9134883

[ref131] Ronit A. Berg R. M. G. Bay J. T. Haugaard A. K. Ahlström M. G. Burgdorf K. S.. (2021). Compartmental immunophenotyping in COVID-19 ARDS: a case series. J. Allergy Clin. Immunol. 147, 81–91. doi: 10.1016/j.jaci.2020.09.009, PMID: 32979342 PMC7581505

[ref132] Rossaint J. Herter J. M. van Aken H. Napirei M. Döring Y. Weber C.. (2014). Synchronized integrin engagement and chemokine activation is crucial in neutrophil extracellular trap-mediated sterile inflammation. Blood 123, 2573–2584. doi: 10.1182/blood-2013-07-516484, PMID: 24335230

[ref133] Sadoff J. Gray G. Vandebosch A. Cárdenas V. Shukarev G. Grinsztejn B.. (2021). Safety and efficacy of single-dose Ad26.COV2.S vaccine against Covid-19. N. Engl. J. Med. 384, 2187–2201. doi: 10.1056/NEJMoa2101544, PMID: 33882225 PMC8220996

[ref134] Saha B. Jyothi Prasanna S. Chandrasekar B. Nandi D. (2010). Gene modulation and immunoregulatory roles of interferon gamma. Cytokine 50, 1–14. doi: 10.1016/j.cyto.2009.11.021, PMID: 20036577

[ref135] Sanche S. Lin Y. T. Xu C. Romero-Severson E. Hengartner N. Ke R. (2020). High contagiousness and rapid spread of severe acute respiratory syndrome coronavirus 2. Emerg. Infect. Dis. 26, 1470–1477. doi: 10.3201/eid2607.200282, PMID: 32255761 PMC7323562

[ref136] Satapathy P. Kumar P. Gupta J. K. Rabaan A. A. al Kaabi N. A. Mohanty D.. (2024). The emergence and implications of SARS-CoV-2 omicron subvariant BA.2.86 on global health. Int. J. Surg. 110, 2498–2501. doi: 10.1097/JS9.0000000000001070, PMID: 38215252 PMC11020040

[ref137] Schneider W. M. Chevillotte M. D. Rice C. M. (2014). Interferon-stimulated genes: a complex web of host defenses. Annu. Rev. Immunol. 32, 513–545. doi: 10.1146/annurev-immunol-032713-120231, PMID: 24555472 PMC4313732

[ref138] Schurink B. Roos E. Radonic T. Barbe E. Bouman C. S. C. de Boer H. H.. (2020). Viral presence and immunopathology in patients with lethal COVID-19: a prospective autopsy cohort study. Lancet Microbe. 1, e290–e299. doi: 10.1016/S2666-5247(20)30144-0, PMID: 33015653 PMC7518879

[ref139] Seppälä E. Veneti L. Starrfelt J. Danielsen A. S. Bragstad K. Hungnes O.. (2021). Vaccine effectiveness against infection with the Delta (B.1.617.2) variant, Norway, April to august 2021. Euro Surveill. 26:793. doi: 10.2807/1560-7917.ES.2021.26.35.2100793, PMID: 34477054 PMC8414959

[ref140] Shang J. Wan Y. Luo C. Ye G. Geng Q. Auerbach A.. (2020). Cell entry mechanisms of SARS-CoV-2. Proc. Natl. Acad. Sci. U. S. A. 117, 11727–11734. doi: 10.1073/pnas.2003138117, PMID: 32376634 PMC7260975

[ref141] Shen Y. Ai J. Lin N. Zhang H. Li Y. Wang H.. (2022). An open, prospective cohort study of VV116 in Chinese participants infected with SARS-CoV-2 omicron variants. Emerg Microbes Infect. 11, 1518–1523. doi: 10.1080/22221751.2022.2078230, PMID: 35579892 PMC9176639

[ref142] Shereen M. A. Khan S. Kazmi A. Bashir N. Siddique R. (2020). COVID-19 infection: origin, transmission, and characteristics of human coronaviruses. J. Adv. Res. 24, 91–98. doi: 10.1016/j.jare.2020.03.005, PMID: 32257431 PMC7113610

[ref143] Shrestha L. B. Foster C. Rawlinson W. Tedla N. Bull R. A. (2022). Evolution of the SARS-CoV-2 omicron variants BA.1 to BA.5: implications for immune escape and transmission. Rev. Med. Virol. 32:e2381. doi: 10.1002/rmv.2381, PMID: 35856385 PMC9349777

[ref144] Smyth D. S. Trujillo M. Gregory D. A. Cheung K. Gao A. Graham M.. (2022). Tracking cryptic SARS-CoV-2 lineages detected in NYC wastewater. Nat. Commun. 13:635. doi: 10.1038/s41467-022-28246-3, PMID: 35115523 PMC8813986

[ref145] Somers E. C. Eschenauer G. A. Troost J. P. Golob J. L. Gandhi T. N. Wang L.. (2020). Tocilizumab for treatment of mechanically ventilated patients with COVID-19. medRxiv [Preprint]. 10.1093/cid/ciaa954 PMC7454462 32651997

[ref146] South A. M. Brady T. M. Flynn J. T. (2020). ACE2 (angiotensin-converting enzyme 2), COVID-19, and ACE inhibitor and Ang II (angiotensin II) receptor blocker use during the pandemic: the Pediatric perspective. Hypertension 76, 16–22. doi: 10.1161/HYPERTENSIONAHA.120.15291, PMID: 32367746 PMC7289676

[ref147] Sui J. Deming M. Rockx B. Liddington R. C. Zhu Q. K. Baric R. S.. (2014). Effects of human anti-spike protein receptor binding domain antibodies on severe acute respiratory syndrome coronavirus neutralization escape and fitness. J. Virol. 88, 13769–13780. doi: 10.1128/JVI.02232-14, PMID: 25231316 PMC4248992

[ref148] Suzuki R. Yamasoba D. Kimura I. Wang L. Kishimoto M. Ito J.. (2022). Attenuated fusogenicity and pathogenicity of SARS-CoV-2 omicron variant. Nature 603, 700–705. doi: 10.1038/s41586-022-04462-1, PMID: 35104835 PMC8942852

[ref149] Tang N. Bai H. Chen X. Gong J. Li D. Sun Z. (2020a). Anticoagulant treatment is associated with decreased mortality in severe coronavirus disease 2019 patients with coagulopathy. J. Thromb. Haemost. 18, 1094–1099. doi: 10.1111/jth.14817, PMID: 32220112 PMC9906401

[ref150] Tang P. Hasan M. R. Chemaitelly H. Yassine H. M. Benslimane F. M. al Khatib H. A.. (2021). BNT162b2 and mRNA-1273 COVID-19 vaccine effectiveness against the SARS-CoV-2 Delta variant in Qatar. Nat. Med. 27, 2136–2143. doi: 10.1038/s41591-021-01583-4, PMID: 34728831

[ref151] Tang N. Li D. Wang X. Sun Z. (2020b). Abnormal coagulation parameters are associated with poor prognosis in patients with novel coronavirus pneumonia. J. Thromb. Haemost. 18, 844–847. doi: 10.1111/jth.14768, PMID: 32073213 PMC7166509

[ref152] Tay M. Z. Poh C. M. Rénia L. MacAry P. A. Ng L. F. P. (2020). The trinity of COVID-19: immunity, inflammation and intervention. Nat. Rev. Immunol. 20, 363–374. doi: 10.1038/s41577-020-0311-8, PMID: 32346093 PMC7187672

[ref153] Teuwen L. A. Geldhof V. Pasut A. Carmeliet P. (2020). COVID-19: the vasculature unleashed. Nat. Rev. Immunol. 20, 389–391. doi: 10.1038/s41577-020-0343-0, PMID: 32439870 PMC7240244

[ref154] Thakur V. Ratho R. K. (2022). OMICRON (B.1.1.529): a new SARS-CoV-2 variant of concern mounting worldwide fear. J. Med. Virol. 94, 1821–1824. doi: 10.1002/jmv.27541, PMID: 34936120

[ref155] The COVID-19 Genomics UK (COG-UK) consortium Volz E. Mishra S. Chand M. Barrett J. C. Johnson R.. (2021). Assessing transmissibility of SARS-CoV-2 lineage B.1.1.7 in England. Nature 593, 266–269. doi: 10.1038/s41586-021-03470-x, PMID: 33767447

[ref156] Tian D. Sun Y. Zhou J. Ye Q. (2022). The global epidemic of SARS-CoV-2 variants and their mutational immune escape. J. Med. Virol. 94, 847–857. doi: 10.1002/jmv.27376, PMID: 34609003 PMC8661756

[ref157] To KK Tsang O. T. Leung W. S. Tam A. R. Wu T. C. Lung D. C.. (2020). Temporal profiles of viral load in posterior oropharyngeal saliva samples and serum antibody responses during infection by SARS-CoV-2: an observational cohort study. Lancet Infect. Dis. 20, 565–574. doi: 10.1016/S1473-3099(20)30196-1, PMID: 32213337 PMC7158907

[ref158] Ulloa A. C. Buchan S. A. Daneman N. Brown K. A. (2022). Estimates of SARS-CoV-2 omicron variant severity in Ontario, Canada. JAMA 327, 1286–1288. doi: 10.1001/jama.2022.2274, PMID: 35175280 PMC8855311

[ref159] van Doremalen N. Purushotham J. N. Schulz J. E. Holbrook M. G. Bushmaker T. Carmody A.. (2021). Intranasal ChAdOx1 nCoV-19/AZD1222 vaccination reduces viral shedding after SARS-CoV-2 D614G challenge in preclinical models. Sci. Transl. Med. 13:755. doi: 10.1126/scitranslmed.abh0755 PMC9267380 34315826

[ref160] van Eijk L. E. Binkhorst M. Bourgonje A. R. Offringa A. K. Mulder D. J. Bos E. M.. (2021). COVID-19: immunopathology, pathophysiological mechanisms, and treatment options. J. Pathol. 254, 307–331. doi: 10.1002/path.5642, PMID: 33586189 PMC8013908

[ref161] VanBlargan L. A. Errico J. M. Halfmann P. J. Zost S. J. Crowe J. E. Jr. Purcell L. A.. (2022). An infectious SARS-CoV-2 B.1.1.529 omicron virus escapes neutralization by therapeutic monoclonal antibodies. Nat. Med. 28, 490–495. doi: 10.1038/s41591-021-01678-y, PMID: 35046573 PMC8767531

[ref162] Varga Z. Flammer A. J. Steiger P. Haberecker M. Andermatt R. Zinkernagel A. S.. (2020). Endothelial cell infection and endotheliitis in COVID-19. Lancet 395, 1417–1418. doi: 10.1016/S0140-6736(20)30937-5, PMID: 32325026 PMC7172722

[ref163] Verdecchia P. Cavallini C. Spanevello A. Angeli F. (2020). The pivotal link between ACE2 deficiency and SARS-CoV-2 infection. Eur. J. Intern. Med. 76, 14–20. doi: 10.1016/j.ejim.2020.04.037, PMID: 32336612 PMC7167588

[ref164] Walls A. C. Park Y. J. Tortorici M. A. Wall A. McGuire A. T. Veesler D. (2020). Structure, function, and antigenicity of the SARS-CoV-2 spike glycoprotein. Cell 181, 281–292. doi: 10.1016/j.cell.2020.02.058, PMID: 32155444 PMC7102599

[ref165] Wang M. Cao R. Zhang L. Yang X. Liu J. Xu M.. (2020). Remdesivir and chloroquine effectively inhibit the recently emerged novel coronavirus (2019-nCoV) in vitro. Cell Res. 30, 269–271. doi: 10.1038/s41422-020-0282-0, PMID: 32020029 PMC7054408

[ref166] Wang W. Yin S. (2024). COVID-19 Hemiencephalitis: A Unique Manifestation. Radiology 310:e231716. doi: 10.1148/radiol.231716, PMID: 38165247

[ref167] Wauters E. van Mol P. Garg A. D. Jansen S. van Herck Y. Vanderbeke L.. (2021). Discriminating mild from critical COVID-19 by innate and adaptive immune single-cell profiling of bronchoalveolar lavages. Cell Res. 31, 272–290. doi: 10.1038/s41422-020-00455-9, PMID: 33473155 PMC8027624

[ref168] Wei C. Shan K. J. Wang W. Zhang S. Huan Q. Qian W. (2021). Evidence for a mouse origin of the SARS-CoV-2 omicron variant. J. Genet. Genomics 48, 1111–1121. doi: 10.1016/j.jgg.2021.12.003, PMID: 34954396 PMC8702434

[ref169] Weisblum Y. Schmidt F. Zhang F. DaSilva J. Poston D. Lorenzi J. C.. (2020). Escape from neutralizing antibodies by SARS-CoV-2 spike protein variants. eLife 9:312. doi: 10.7554/eLife.61312 PMC7723407 33112236

[ref170] Wen W. Chen C. Tang J. Wang C. Zhou M. Cheng Y.. (2022). Efficacy and safety of three new oral antiviral treatment (molnupiravir, fluvoxamine and Paxlovid) for COVID-19:a meta-analysis. Ann. Med. 54, 516–523. doi: 10.1080/07853890.2022.2034936, PMID: 35118917 PMC8820829

[ref171] Wolter N. Jassat W. Walaza S. Welch R. Moultrie H. Groome M.. (2022). Early assessment of the clinical severity of the SARS-CoV-2 omicron variant in South Africa: a data linkage study. Lancet 399, 437–446. doi: 10.1016/S0140-6736(22)00017-4, PMID: 35065011 PMC8769664

[ref172] Wong L. R. Perlman S. (2022). Immune dysregulation and immunopathology induced by SARS-CoV-2 and related coronaviruses—are we our own worst enemy? Nat. Rev. Immunol. 22, 47–56. doi: 10.1038/s41577-021-00656-2, PMID: 34837062 PMC8617551

[ref173] Wrapp D. Wang N. Corbett K. S. Goldsmith J. A. Hsieh C. L. Abiona O.. (2020). Cryo-EM structure of the 2019-nCoV spike in the prefusion conformation. Science 367, 1260–1263. doi: 10.1126/science.abb2507, PMID: 32075877 PMC7164637

[ref174] Wu Y. Shen Y. Wu N. Zhang X. Chen S. Yang C.. (2022). Omicron-specific mRNA vaccine elicits potent immune responses in mice, hamsters, and nonhuman primates. Cell Res. 32, 949–952. doi: 10.1038/s41422-022-00706-x, PMID: 35915244 PMC9340695

[ref175] Xia S. Yan L. Xu W. Agrawal A. S. Algaissi A. Tseng C. K.. (2019). A pan-coronavirus fusion inhibitor targeting the HR1 domain of human coronavirus spike. Sci. Adv. 5:4580. doi: 10.1126/sciadv.aav4580 PMC6457931 30989115

[ref176] Xu Z. Shi L. Wang Y. Zhang J. Huang L. Zhang C.. (2020). Pathological findings of COVID-19 associated with acute respiratory distress syndrome. Lancet Respir. Med. 8, 420–422. doi: 10.1016/S2213-2600(20)30076-X, PMID: 32085846 PMC7164771

[ref177] Xu S. Yang K. Li R. Zhang L. (2020). mRNA vaccine era-mechanisms drug platform and clinical prospection. Int. J. Mol. Sci. 21:582. doi: 10.3390/ijms21186582, PMID: 32916818 PMC7554980

[ref178] Yan R. Zhang Y. Li Y. Xia L. Guo Y. Zhou Q. (2020). Structural basis for the recognition of SARS-CoV-2 by full-length human ACE2. Science 367, 1444–1448. doi: 10.1126/science.abb2762, PMID: 32132184 PMC7164635

[ref179] Yang X. Yu Y. Xu J. Shu H. Xia J. Liu H.. (2020). Clinical course and outcomes of critically ill patients with SARS-CoV-2 pneumonia in Wuhan, China: a single-centered, retrospective, observational study. Lancet Respir. Med. 8, 475–481. doi: 10.1016/S2213-2600(20)30079-5, PMID: 32105632 PMC7102538

[ref180] You J. Dove B. K. Enjuanes L. DeDiego M. L. Alvarez E. Howell G.. (2005). Subcellular localization of the severe acute respiratory syndrome coronavirus nucleocapsid protein. J. Gen. Virol. 86, 3303–3310. doi: 10.1099/vir.0.81076-0, PMID: 16298975

[ref181] Yuan Y. Cao D. Zhang Y. Ma J. Qi J. Wang Q.. (2017). Cryo-EM structures of MERS-CoV and SARS-CoV spike glycoproteins reveal the dynamic receptor binding domains. Nat. Commun. 8:15092. doi: 10.1038/ncomms15092, PMID: 28393837 PMC5394239

[ref182] Zandi M. (2022). ORF9c and ORF10 as accessory proteins of SARS-CoV-2 in immune evasion. Nat. Rev. Immunol. 22:331. doi: 10.1038/s41577-022-00715-2, PMID: 35361899 PMC8970066

[ref183] Zandi M. Shafaati M. Kalantar-Neyestanaki D. Pourghadamyari H. Fani M. Soltani S.. (2022). The role of SARS-CoV-2 accessory proteins in immune evasion. Biomed. Pharmacother. 156:113889. doi: 10.1016/j.biopha.2022.113889, PMID: 36265309 PMC9574935

[ref184] Zazhytska M. Kodra A. Hoagland D. A. Frere J. Fullard J. F. Shayya H.. (2022). Non-cell-autonomous disruption of nuclear architecture as a potential cause of COVID-19-induced anosmia. Cell 185, 1052–1064. doi: 10.1016/j.cell.2022.01.024, PMID: 35180380 PMC8808699

[ref185] Zhang Y. Z. Holmes E. C. (2020). A genomic perspective on the origin and emergence of SARS-CoV-2. Cell 181, 223–227. doi: 10.1016/j.cell.2020.03.035, PMID: 32220310 PMC7194821

[ref186] Zhang W. Huang L. Ye G. Geng Q. Ikeogu N. Harris M.. (2022). Vaccine booster efficiently inhibits entry of SARS-CoV-2 omicron variant. Cell. Mol. Immunol. 19, 445–446. doi: 10.1038/s41423-022-00837-6, PMID: 35075267 PMC8785151

[ref187] Zhang Y. Juhas M. Kwok C. K. (2023). Aptamers targeting SARS-COV-2: a promising tool to fight against COVID-19. Trends Biotechnol. 41, 528–544. doi: 10.1016/j.tibtech.2022.07.012, PMID: 35995601 PMC9340053

[ref188] Zhang H. Penninger J. M. Li Y. Zhong N. Slutsky A. S. (2020). Angiotensin-converting enzyme 2 (ACE2) as a SARS-CoV-2 receptor: molecular mechanisms and potential therapeutic target. Intensive Care Med. 46, 586–590. doi: 10.1007/s00134-020-05985-9, PMID: 32125455 PMC7079879

[ref189] Zhang X. Wu S. Wu B. Yang Q. Chen A. Li Y.. (2021). SARS-CoV-2 omicron strain exhibits potent capabilities for immune evasion and viral entrance. Signal Transduct. Target. Ther. 6:430. doi: 10.1038/s41392-021-00852-5, PMID: 34921135 PMC8678971

[ref190] Zhang Y. Xiao M. Zhang S. Xia P. Cao W. Jiang W.. (2020). Coagulopathy and antiphospholipid antibodies in patients with Covid-19. N. Engl. J. Med. 382:e38. doi: 10.1056/NEJMc2007575, PMID: 32268022 PMC7161262

[ref191] Zhao H. To KKW Lam H. Zhou X. Chan J. F. Peng Z.. (2021). Cross-linking peptide and repurposed drugs inhibit both entry pathways of SARS-CoV-2. Nat. Commun. 12:1517. doi: 10.1038/s41467-021-21825-w, PMID: 33750821 PMC7943568

[ref192] Zheng C. Shao W. Chen X. Zhang B. Wang G. Zhang W. (2022). Real-world effectiveness of COVID-19 vaccines: a literature review and meta-analysis. Int. J. Infect. Dis. 114, 252–260. doi: 10.1016/j.ijid.2021.11.009, PMID: 34800687 PMC8595975

[ref193] Zheng H. Y. Zhang M. Yang C. X. Zhang N. Wang X. C. Yang X. P.. (2020). Elevated exhaustion levels and reduced functional diversity of T cells in peripheral blood may predict severe progression in COVID-19 patients. Cell. Mol. Immunol. 17, 541–543. doi: 10.1038/s41423-020-0401-3, PMID: 32203186 PMC7091621

[ref194] Zhou P. Yang X. L. Wang X. G. Hu B. Zhang L. Zhang W.. (2020). A pneumonia outbreak associated with a new coronavirus of probable bat origin. Nature 579, 270–273. doi: 10.1038/s41586-020-2012-7, PMID: 32015507 PMC7095418

